# Real-World Body Composition and Blood Pressure Changes Following Glucagon-Like Peptide 1 Receptor Agonist Initiation: A Causal Inference Study of Connected Device Data

**DOI:** 10.1016/j.mcpdig.2026.100375

**Published:** 2026-07-28

**Authors:** Agathe Chabassier, Benjamin Vittrant, Pierre Binon, Erwan Scornet, Julie Josse

**Affiliations:** a Sorbonne University, CNRS, Laboratory of Probability Statistics and Modelling (LPSM), Paris, France; b Inria, Inserm, PreMeDICaL Team, University of Montpellier, France; c Withings, Issy-les-Moulineaux, France

## Abstract

**Objective:**

To characterize real-world body composition and blood pressure changes following glucagon-like peptide (GLP)-1 receptor agonist (RA) initiation and identify predictors of weight loss quality using connected device data.

**Patients and Methods:**

In this retrospective cohort study, adult US users completing an in-application GLP-1 RA survey (June 8, 2023, to July 4, 2024) were followed up using longitudinal device data (December 2021 to September 2025). We used a distributional causal inference framework to match GLP-1 RA users to nonusers. Primary estimands were the distributional average treatment effect on the treated and median differences over months 10 to 14.

**Results:**

Among 2031 users for body composition (396 exposed) and 672 for blood pressure (BP; 148 exposed), GLP-1 RA initiation was associated with a 9.8% median weight reduction (95% CI, 9.8-9.9), driven by fat mass loss (7.5%; 95% CI, 7.5-7.8) with smaller muscle mass loss (2.3%; 95% CI, 2.1-2.4). Sustained reductions were observed in systolic (2.5 mm Hg; 95% CI, 1.5-2.9) and diastolic BP (1.5 mm Hg; 95% CI, 1.2-1.9). Baseline muscle-to-fat ratio (MFR) was the strongest predictor of weight loss quality, with lower MFR individuals losing more fat and sparing more muscle.

**Conclusion:**

In a real-world setting, GLP-1 RA initiation was associated with clinically significant fat-predominant weight loss and sustained BP improvements. Baseline MFR predicted weight loss quality, suggesting that pretreatment body composition may help clinicians anticipate individual responses—a step toward precision medicine in GLP-1 RA therapy.

Glucagon-like peptide-1 receptor agonists (GLP-1 RAs) have significantly altered the management of obesity and cardiovascular risk. Landmark randomized controlled trials, including the Semaglutide Treatment Effect in People with obesity (STEP) program for semaglutide^1^ and the Semaglutide Effects on Cardiovascular Outcomes in People with Overweight or Obesity (SELECT) trial,^2^ showed substantial weight loss and cardiovascular benefits. However, strict inclusion criteria and standardized titration in trials do not always reflect routine clinical practice, creating an efficacy-effectiveness gap driven by variable adherence, unmonitored titration, and gastrointestinal adverse events.^3^

However, the physiological quality of weight loss in the real-world remains undercharacterized. While trials suggest a favorable body composition profile, concerns persist regarding sarcopenia and muscle preservation in broader, less active populations outside of trial settings.^4^^,^^5^ Real-world evidence suggests that tissue remodeling is highly dynamic, often involving an initial catabolic phase followed by stabilization.^6^ However, the use of sparse or aggregated data often precludes the robust analysis of temporal dynamics; furthermore, the frequent absence of control groups for counterfactual comparison makes it difficult to distinguish causal drug effects from natural physiological fluctuations or regression to the mean.^6^^,^^7^ Temporal dynamics of blood pressure (BP) changes, specifically whether reductions are sustained amidst daily fluctuations, are also difficult to capture with sparse clinical measurements.^8^

Connected health devices, such as smart scales and BP monitors, generate high-frequency time-series data capable of investigating these dynamics.^9^ However, leveraging this density requires suitable methodologies. Standard observational analyses often compress longitudinal history into scalar averages (eg, mean pretreatment weight), obscuring physiological volatility.^10^ To address this, we used a distributional causal inference framework, Adaptive Distributional Matching with Local 2-Stage (ADD-MALTS),^11^ which matches users based on their full pretreatment outcome distribution. We characterized the real-world variations in body composition and BP following GLP-1 RA initiation in a large cohort of connected device users, with particular attention to the heterogeneity of treatment response across baseline physiological profiles.

## Patients And Methods

### Study Design and Participants

We conducted a retrospective observational cohort study using longitudinal data from Withings connected health devices (smart scales and BP monitors). The study was conducted in strict adherence to the General Data Protection Regulation and Health Insurance Portability and Accountability Act security standards. All participants provided digital informed consent for the use of their data for research purposes via the Withings application.

The source population comprised adult users in the United States who satisfied the following inclusion criteria: (1) completion of an in-application GLP-1 RA survey between June 8, 2023, and July 4, 2024; (2) possession of sufficient longitudinal data, requiring at least 1 valid measurement in each of 3 temporal windows relative to the index date (*t*_0_): a baseline history (−6 to −1 months), an induction phase (0-9 months), and an efficacy window (+10 to +14 months); (3) age between 22 and 80 years; (4) baseline body mass index (BMI) between 10 and 60 kg/m^2^; and (5) initiation of GLP-1 RA therapy no more than 12 months before the survey. To satisfy the requirements for a 6-month baseline history and a 14-month postinitiation follow-up, the total study period included longitudinal data recorded from December 2021 through September 2025. This selection process yielded 2 distinct analytic samples based on measurement availability: cohort 1 (body composition; n=2031) and cohort 2 (BP; n=672).

Data regarding race and ethnicity were not collected by the connected devices and were therefore not available for analysis; we discuss the implications of this potential unmeasured confounding in the Discussion section. User sex was self-reported as a binary category (male/female) during device setup and included as a baseline covariate in the matching process. As sex assigned at birth was not separately collected, this variable is used as a proxy for both biological sex attributes and gender identity.

### Procedures and Outcomes

For unexposed users, the index date (*t*_0_) was the survey date. For exposed users, *t*_0_ was imputed as the rounded midpoint of the self-reported treatment duration interval (eg, 5 months before survey for the 3-6 months’ category) (Supplemental Appendix 1, available online at https://www.mcpdigitalhealth.org/). Users who reported initiating GLP-1 RA therapy more than one year before the survey were excluded to ensure the accuracy of the imputed start date.

Outcomes were processed as daily means to remove noise from repeated same-day measurements while preserving day-to-day physiological volatility. We conducted separate distributional causal inference analyses for each cohort as follows:

1. Cohort 1 (body composition): The outcomes of interest were total weight, device-estimated fat mass, and device-estimated muscle mass. Total weight was measured via high-precision load cells. Body composition was estimated using bioelectrical impedance analysis (BIA), which distinguishes fat mass from fat-free mass. Fat-free mass (commonly referred to as lean mass) comprises bone mineral content and muscle mass (including body water). Since bone mineral content is assumed stable over 14 months, longitudinal changes in fat-free mass are driven primarily by changes in muscle mass. Our device-estimated muscle mass therefore tracks the same compartment as the lean mass changes reported in clinical trial literature. Importantly, BIA is sensitive to hydration and any water loss (dehydration) is attributed to muscle loss by the algorithm. However, as GLP-1 RAs may induce dehydration rather than the contrary,^3^ our estimates provide a conservative upper bound for muscle loss.
2. Cohort 2 (BP): The outcomes of interest were systolic and diastolic BP, obtained via clinically validated monitors.^12^^,^^13^

Measurements were initiated spontaneously by the user without digital prompts or incentives, by simply stepping onto the scale or pressing a button on the BP cuff. The devices use historical data to automatically recognize and assign measurements to the correct user profile. Body composition data were collected using 5 Withings smart scale models (body plus, body smart, body cardio, body comp, and body scan), all sharing a standardized multielectrode BIA platform for weight and body composition estimation. Blood pressure data were primarily captured by the BPM connect, a clinically validated oscillometric monitor. A detailed description of these devices, their technical specifications, and the hardware distribution across the study groups is provided in Supplemental Appendix 1, available online at https://www.mcpdigitalhealth.org/.

### Statistical Analyses

The primary causal estimand was the average treatment effect on the treated represented by distributions, which we term distributional average treatment effect on the treated during the 1-year efficacy window (months 10-14). To account for physiological volatility masked by scalar averages, we used the ADD-MALTS framework^11^ (Supplemental Appendix 1, available online at https://www.mcpdigitalhealth.org/). This approach matches users based on static covariates (age, self-reported gender, BMI, and baseline physical activity) and their full 6-month pretreatment outcome distribution (*t*-6 to *t*-1). Exposed users were matched to unexposed controls using adaptive nearest-neighbor matching (*k*=5). The analysis was implemented in Python using the pyaddmalts package (available at https://github.com/almost-matching-exactly/addmalts).

To evaluate the 1-year results, we reconstructed body composition and BP distributions using the data points recorded during the 10-14-month primary efficacy window. This 4-month interval, centered at 12 months, balances temporal precision with sufficient data density to reconstruct outcome distributions (detailed justification in Supplemental Appendix 1, available online at https://www.mcpdigitalhealth.org/).

We estimated the difference between the medians of the averaged exposed and counterfactual distributions, which we call scalar average treatment effect on the treated (sATT). Uncertainty was assessed using 500 bootstrap replicates. Within each replicate, the distance metric was trained on a 20% subsample, while matching and effect estimation were performed on the remaining users (Supplemental Appendix 1, available online at https://www.mcpdigitalhealth.org/). Longitudinal trajectories were reconstructed by applying functional data analysis via Principal component Analysis through Conditional Expectation to the matched cohorts, allowing for the reconstruction of smooth trajectories from sparse discrete measurements.^14^^,^^15^ Reported 95% CIs reflect pointwise uncertainty.^16^^,^^17^

The scalar effects (sATT) were assessed on subgroups defined by age, self-reported gender, baseline BMI, and baseline muscle-to-fat ratio (MFR). We refer to these subgroup effect estimates as scalar conditional average treatment effect on the treated (sCATT). Additionally, to identify the main drivers of heterogeneity of treatment effects among these correlated variables, we used a bootstrap-based best linear projection procedure.^18^ For each variable, we report both subgroup-specific sCATT and linear projection coefficients to offer a detailed view of heterogeneity (Supplemental Appendix 1, available online at https://www.mcpdigitalhealth.org/).

Sensitivity analyses included varying the number of neighbors and the index date definition. Two supportive parametric models, event-study linear mixed-effects model (LMEM) and fixed effects (FEs) were specified to triangulate findings (Supplemental Methods [p 2], available online at https://www.mcpdigitalhealth.org/). LMEM and FE models, which assume linear parametric relationships, served as sensitivity checks against the nonparametric ADD-MALTS framework (Supplemental Appendix 1, available online at https://www.mcpdigitalhealth.org/).

## Results

### Study Population and Matching Quality

**Figure 1:**
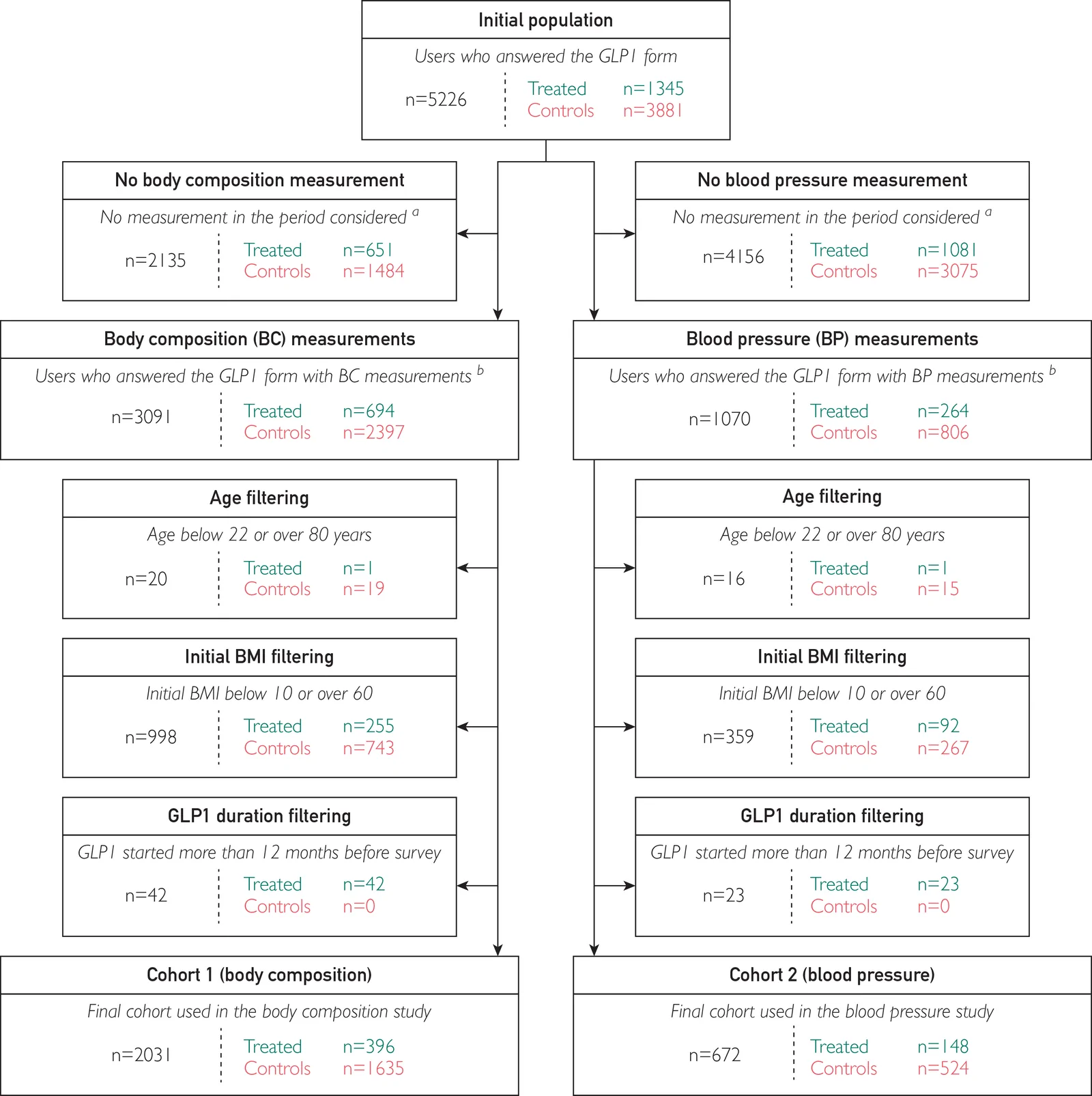
Study flowchart and cohort formation. Flow diagram illustrating the selection process for the final analytic cohorts from the initial survey population. The diagram details the exclusion criteria applied sequentially. Two distinct analytic samples were derived: cohort 1 (n=2031) for the analysis of body composition outcomes and cohort 2 (n=672) for the analysis of blood pressure outcomes. ^a^Defined as the requirement for at least 1 measurement in each of the 3 key temporal windows: the preinclusion history (6 months before *t*_0_ for adjustment), the active study period (10 months following *t*_0_), and the 1-year efficacy window (months 10-14 postinitiation). ^b^Refers to users possessing valid measurements in all 3 periods specified above.

As illustrated in our population flowchart (Figure 1), we identified 5226 Withings users who responded to the GLP-1 RA survey (1345 self-reported GLP-1 RA takers; 3881 nontakers). The primary reason for exclusion was the lack of measurement density in the required temporal windows (n=2135 for body composition; n=4156 for BP). Additional exclusions were made for age (n=20 for body composition; n=16 for BP), initial BMI (n=998 for body composition; n=359 for BP), and treatment duration exceeding 12 months (n=42 for body composition; n=23 for BP). This yielded a final sample of 396 exposed users and 1635 matched controls for cohort 1 and 148 exposed users and 524 matched controls for cohort 2.

Before matching, exposed users were older, had higher baseline BMI, and were less physically active than nonusers (Table, Supplemental Figure 4, available online at https://www.mcpdigitalhealth.org/). The matching procedure substantially improved balance (Supplemental Figure 1, available online at https://www.mcpdigitalhealth.org/ and Supplemental Table 1, available online at https://www.mcpdigitalhealth.org/): in cohort 1, standardized mean differences fell below 0.2 for age, sex, and step count; in cohort 2, a residual imbalance remained for BMI (∼0.3), although the pretreatment outcome distributions were visually superimposable between groups (Figure 4). In a subset of participants with available supplementary health data (∼30%), the prevalence of type 2 diabetes was low (3%-4%), suggesting the cohort is primarily composed of individuals engaged in weight management.

**Table:** Baseline Characteristics of the Study Population

Characteristic	Cohort 1 (body composition)			Cohort 2 (blood pressure)		
	With GLP1 intake (n=396)	Without GLP1 intake (n=1635)	Overall (n=2031)	With GLP1 intake (n=148)	Without GLP1 intake (n=524)	Overall (n=672)
Age (y)						
18-39	58 (14.6)	359 (22.0)	417 (20.5)	11 (7.4)	56 (10.7)	67 (10.0)
40-59	250 (63.1)	950 (58.1)	1200 (59.1)	95 (64.2)	276 (52.7)	371 (55.2)
60+	88 (22.2)	326 (19.9)	414 (20.4)	42 (28.4)	192 (36.6)	234 (34.8)
Missing	0 (0.0)	0 (0.0)	0 (0.0)	0 (0.0)	0 (0.0)	0 (0.0)
Initial BMI (kg/m^2^)						
Underweight	1 (0.3)	9 (0.6)	10 (0.5)	0 (0.0)	5 (1.0)	5 (0.7)
Healthy	17 (4.3)	379 (23.2)	396 (19.5)	4 (2.7)	113 (21.6)	117 (17.4)
Overweight	104 (26.3)	638 (39.0)	742 (36.5)	30 (20.3)	211 (40.3)	241 (35.9)
Obesity	274 (69.2)	609 (37.2)	883 (43.5)	114 (77.0)	195 (37.2)	309 (46.0)
Missing	0 (0.0)	0 (0.0)	0 (0.0)	0 (0.0)	0 (0.0)	0 (0.0)
Gender						
Female	180 (45.5)	670 (41.0)	850 (41.9)	46 (31.1)	127 (24.2)	173 (25.7)
Male	216 (54.5)	965 (59.0)	1181 (58.1)	102 (68.9)	397 (75.8)	499 (74.3)
Missing	0 (0.0)	0 (0.0)	0 (0.0)	0 (0.0)	0 (0.0)	0 (0.0)
Initial physical activity						
Inactive (<5000 steps)	158 (39.9)	428 (26.2)	586 (28.9)	53 (35.8)	146 (27.9)	199 (29.6)
Moderately active (5000-9999 steps per day)	196 (49.5)	936 (57.2)	1132 (55.7)	80 (54.1)	284 (54.2)	364 (54.2)
Active (≥10,000 steps per day)	22 (5.6)	241 (14.7)	263 (12.9)	7 (4.7)	83 (15.8)	90 (13.4)
Missing	20 (5.1)	30 (1.8)	50 (2.5)	8 (5.4)	11 (2.1)	19 (2.8)

Data are presented as n (%). The “without GLP1 intake” column displays characteristics of the non–GLP-1 RA user cohort. The “with GLP1 intake column” displays characteristics of the self-reported GLP-1 RA user cohort before matching.

BMI, body mass index; GLP-1 RA, glucagon-like peptide-1 receptor agonist.

Measurement frequencies were comparable between the exposed and unexposed groups for cohorts 1 and 2 (Supplemental Figure 3, available online at https://www.mcpdigitalhealth.org/). Although weight monitoring increased immediately at inclusion (*t*_0_), BP monitoring in the exposed group rose more gradually, reaching a delayed peak median of 2.3 measurements per month during months 3-6. This lag underscores the necessity of our 4-month window (months 10-14) to ensure robust estimation of distributions.

### Body Composition Changes Following GLP-1 RA Initiation

**Figure 2:**
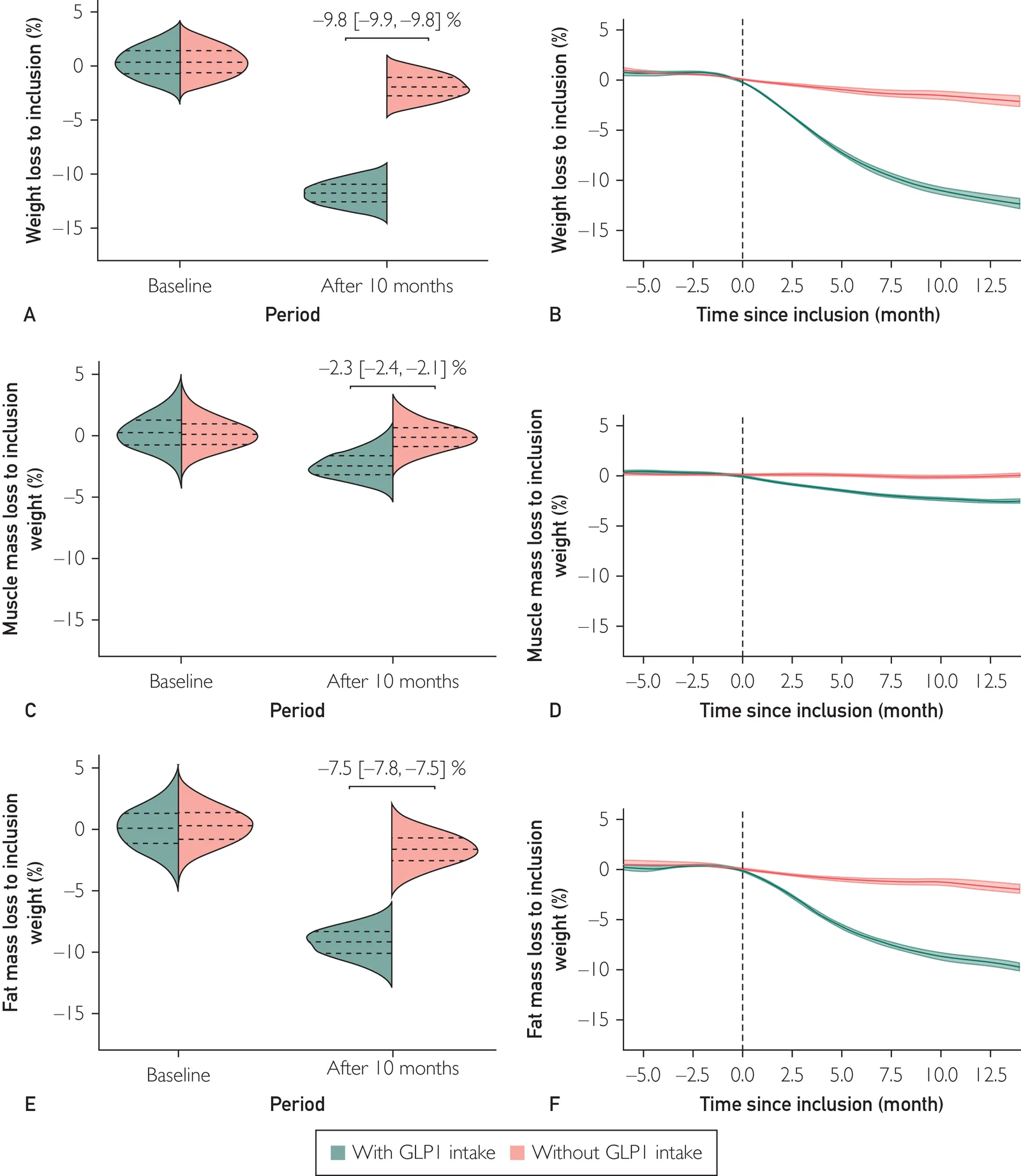
Distributional and longitudinal effects of glucagon-like peptide-1 receptor agonist (GLP-1 RA) initiation on body composition (cohort 1). (A, C, E) Plots displaying the distributions barycenters of values of percentage change in total body weight (A), device-estimated muscle mass (C), and device-estimated fat mass (E) relative to inclusion weight across the baseline period (the preinclusion window of 6 months) and the “after 10 months” 4-month period (the 1-year efficacy window, months 10-14). The left side (green) represents the barycenter distribution in the exposed group, and the right side (red) represents the barycenter distribution in the matched unexposed group (counterfactuals). The bracketed values display the associated scalar average treatment effect on the treated estimate, the estimated difference between the median of the 2 barycenter distributions, with 95% bootstrap CIs. (B, D, F) Longitudinal cross-sectional mean trajectories of percentage change in total body weight (B), device-estimated muscle mass (D), and device-estimated fat mass (F) from 6 months before 14 months postinclusion (*t*_0_). Trajectories were reconstructed using functional data analysis via Principal component Analysis through Conditional Expectation. The green line represents the exposed group, and the red line represents the matched unexposed group (counterfactual). Shaded areas indicate 95% bootstrap pointwise CI.

In the 1-year efficacy window (months 10-14), GLP-1 RA initiation was associated with a median total weight reduction of 9.8% (sATT, −9.8%; 95% CI, −9.9 to −9.8) relative to the counterfactual group (Figure 2). This reduction was driven predominantly by loss of device-estimated fat mass (sATT, −7.5%; 95% CI, −7.8 to −7.5), whereas device-estimated muscle mass decreased by only 2.3% (95% CI, −2.4 to −2.1). In absolute terms, this translated to a median reduction of 10.2 kg in total weight, composed of 7.7 kg lost device-estimated fat mass, and 2.2 kg lost muscle mass (Supplemental Figure 5, available online at https://www.mcpdigitalhealth.org/). Consequently, device-estimated muscle loss accounted for 21.6% (95% CI, 20.6-22.5) of the total weight reduction.

GLP-1 RA initiators also exhibited higher longitudinal retention than matched controls: approximately 40% continued active weighing beyond 24 months, whereas most controls ceased monitoring by month 20 (Supplemental Figure 15, available online at https://www.mcpdigitalhealth.org/).

### Heterogeneity of Weight Loss

**Figure 3:**
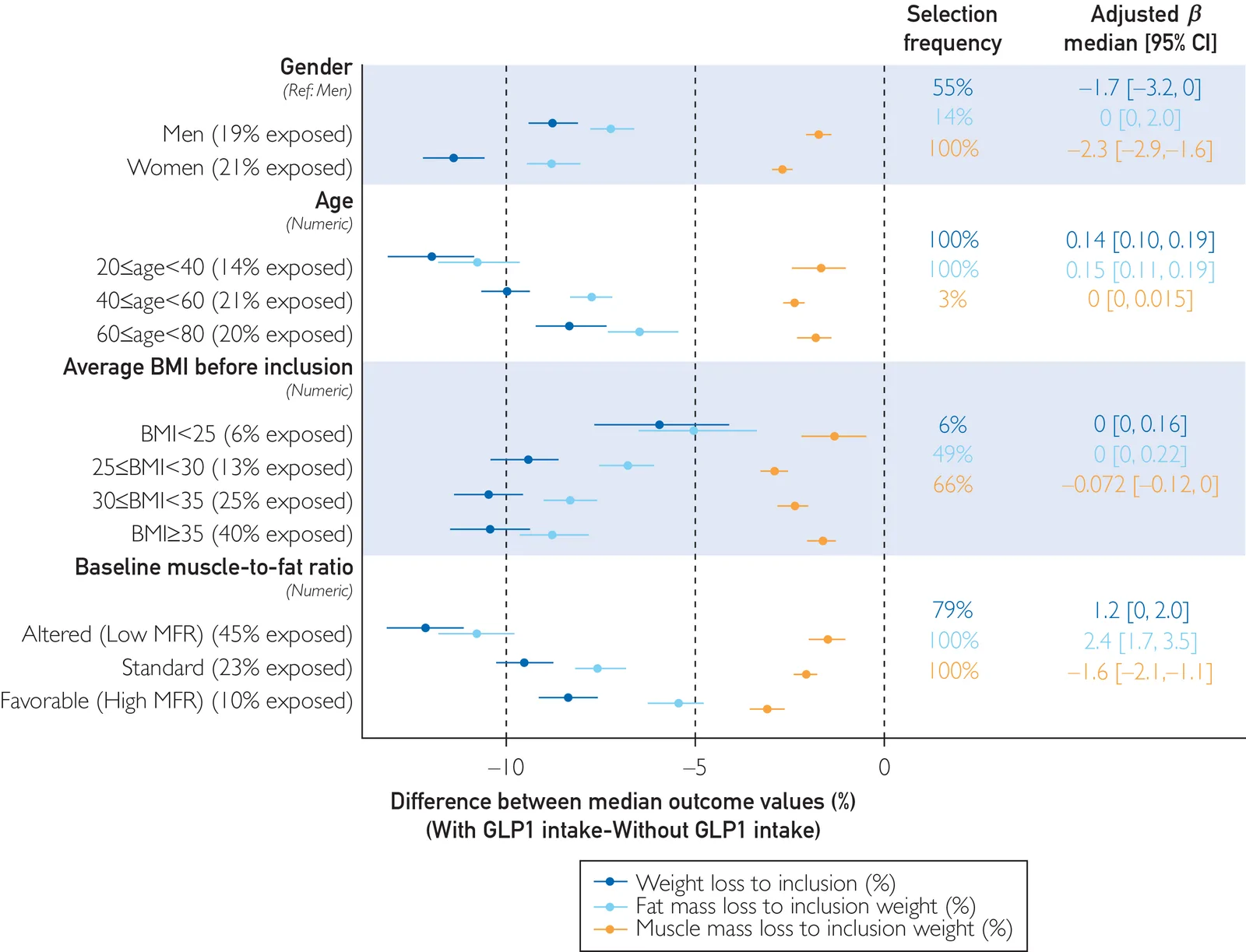
Heterogeneity of treatment effect on body composition by baseline characteristics. Forest plot displaying the scalar conditional average treatment effect on the treated (sCATT) on percentage weight loss, device-estimated fat mass loss, and device-estimated muscle mass loss across stratifications of self-reported gender, age, baseline body mass index (BMI), and baseline muscle-to-fat ratio. Points represent the median difference between the barycenter distributions of the exposed and unexposed outcome distributions within each subgroup. Error bars indicate 95% CIs derived from 500 bootstrap replicates. Selection frequency indicates the proportion of bootstrap replicates in which the variable was retained as a robust independent effect modifier in the multivariable variable selection model. Adjusted β coefficients represent the median linear contribution of each baseline characteristic to the treatment effect size; unlike the stratified sCATT estimates, these coefficients isolate the independent contribution of each variable by accounting for correlations between baseline covariates.

Variable selection identified distinct drivers of treatment response (Figure 3). Baseline BMI was associated with the absolute magnitude of weight loss (Supplemental Figure 7, available online at https://www.mcpdigitalhealth.org/), whereas demographic factors and initial body composition influenced relative efficiency: women, younger adults, and patients with a lower MFR achieved greater percentage weight reductions than men and older users (Supplemental Figure 10, available online at https://www.mcpdigitalhealth.org/).

Among all baseline characteristics examined, the MFR emerged as the strongest and most consistent independent predictor of weight loss quality. It was retained in 100% of bootstrap replicates for both device-estimated fat and muscle mass outcomes. Individuals with a lower baseline MFR (altered body composition) lost more fat (adjusted β, 2.4% per MFR unit; 95% CI, 1.7-3.5) and spared more muscle (adjusted β, −1.6% per MFR unit; 95% CI, −2.1 to −1.1) than those with a favorable profile (Supplemental Figures 11 and 12, available online at https://www.mcpdigitalhealth.org/). In other words, treatment response was most favorable, in terms of body composition remodeling, in the patients with the highest baseline adiposity relative to muscle mass.

### Blood Pressure Changes Following GLP-1 RA Initiation

**Figure 4:**
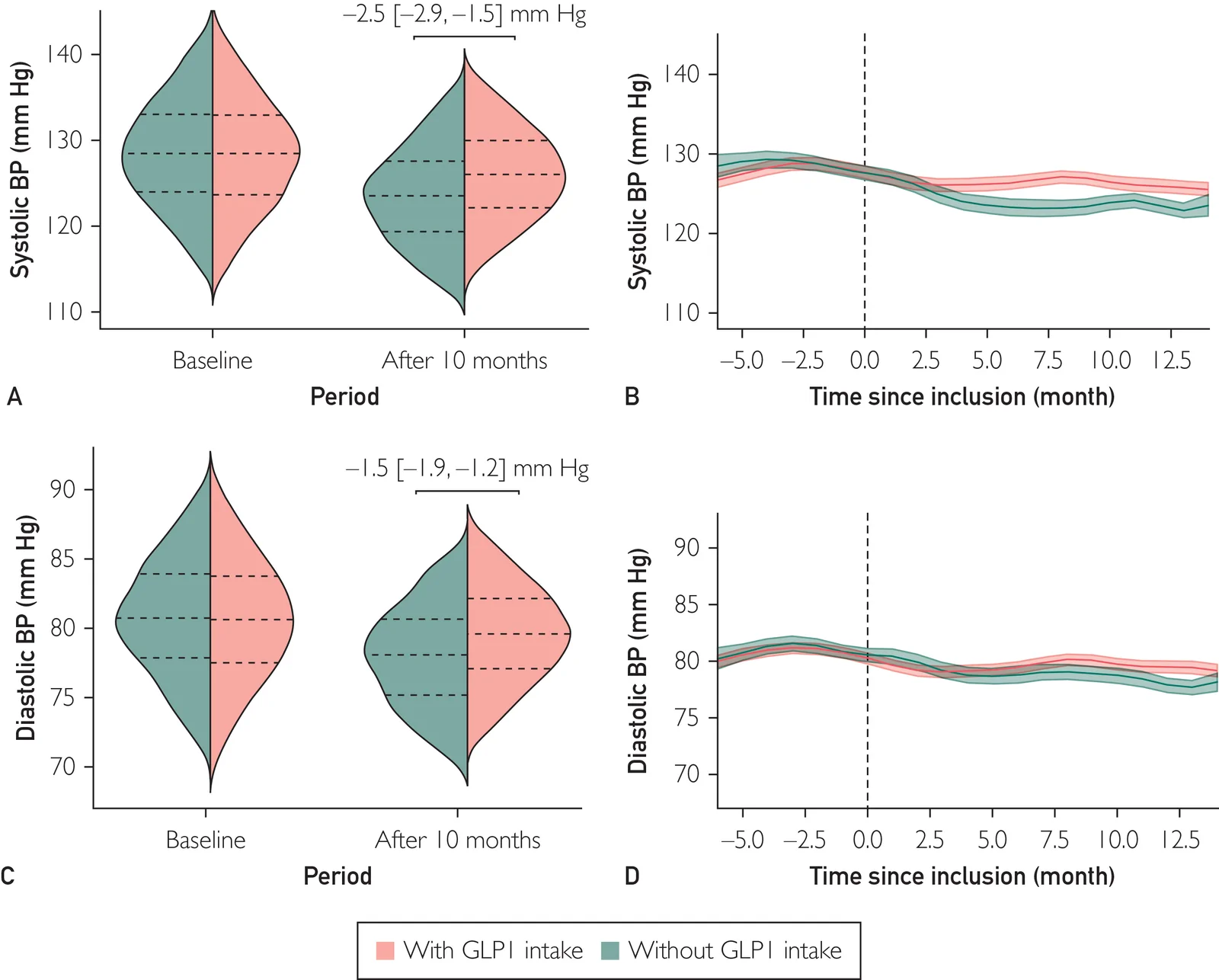
Distributional and longitudinal effects of glucagon-like peptide-1 receptor agonist (GLP-1 RA) initiation on blood pressure (cohort 2). (A, C) Plots displaying the distributions barycenters of values of systolic (A) and diastolic (C) blood pressure (mm Hg) across the baseline period (the preinclusion window of 6 months) and the “after 10 months” 4-month period (the 1-year efficacy window, months 10-14). The left side (green) represents the barycenter distribution in the exposed group, and the right side (red) represents the barycenter distribution in the matched unexposed group (counterfactuals). The bracketed values display the associated scalar average treatment effect on the treated estimate, the estimated difference between the median of the 2 barycenter distributions, with 95% bootstrap CI. (B, D) Longitudinal trajectories of systolic (B) and diastolic (D) blood pressure from 6 months before 14 months postinclusion (*t*_0_), estimated via Principal component Analysis through Conditional Expectation smoothing. The green line represents the exposed group, and the red line represents the matched unexposed group (counterfactual). Shaded areas indicate 95% bootstrap pointwise CI.

GLP-1 RA initiation was associated with sustained BP improvements. Relative to matched controls, exposed users achieved a median reduction of 2.5 mm Hg in systolic BP (sATT, −2.5; 95% CI, −2.9 to −1.5) and 1.5 mm Hg in diastolic pressure (sATT, −1.5; 95% CI, −1.9 to −1.2) (Figure 4). Longitudinal reconstruction revealed that although unexposed users exhibited characteristic seasonal BP increases during winter months, exposed users defied this cyclic rebound, maintaining stable reductions throughout the observation period (Figure 4; Supplemental Figure 8, available online at https://www.mcpdigitalhealth.org/). Stratified analyses did not identify robust independent predictors of BP response, likely owing to limited power in this subcohort (Supplemental Figures 2, 13, and 14, available online at https://www.mcpdigitalhealth.org/).

### Robustness and Sensitivity Analyses

Results were robust to methodological specifications. Comparison of smoothed versus raw longitudinal trajectories confirmed the fidelity of the functional data analysis reconstruction (Supplemental Figures 6 and 9, available online at https://www.mcpdigitalhealth.org/). Sensitivity analyses varying the number of matched neighbors^2^^,^^10^ and shifting the imputed index date (*t*_0_ shifted by 2 months in either direction) yielded consistent estimated differences (Supplemental Appendix 1, available online at https://www.mcpdigitalhealth.org/). Furthermore, supportive parametric models (LMEM and FE) confirmed the directionality of the primary findings, albeit with larger estimated magnitudes (total weight reductions, 11.7%-12.8%; systolic BP reductions, 3.7-4.8 mm Hg) (Supplemental Tables 2 and 3, available online at https://www.mcpdigitalhealth.org/). These larger estimates in linear models highlight the conservative nature of the primary distributional analysis.

## Discussion

### Principal Findings and Digital Engagement Dynamics

In this large-scale retrospective cohort study, we used a distributional causal inference framework to characterize the changes in body composition and BP following GLP-1 RA initiation. By comparing exposed users to a matched counterfactual group, this framework addresses a critical limitation of recent single-arm prospective evaluations, which lack control groups to account for natural physiological drift or regression to the mean.^6^^,^^7^ A technical discussion regarding the adjustment for regression to the mean and the rationale for the nonparametric distributional framework is provided in Supplemental Appendix 1, available online at https://www.mcpdigitalhealth.org/. However, while our approach estimates specific differences associated with therapy by removing some confounding bias, these results represent observed associations and should not be viewed as definitive pharmacologic evidence of the type provided by randomized clinical trials.

We found that initiation of therapy was associated with a median total weight reduction of 9.8% (95% CI, 9.8-9.9) over 1 year. Importantly, this weight loss was characterized by a favorable body composition profile: device-estimated muscle mass loss accounted for only 21.6% (95% CI, 20.6-22.5) of the total reduction, with the majority driven by device-estimated fat mass loss. Concurrently, users achieved sustained reductions in systolic (2.5 mm Hg; 95% CI, 1.5-2.9) and diastolic (1.5 mm Hg; 95% CI, 1.2-1.9) BP that persisted throughout the year and were maintained despite the seasonal increases observed in the unexposed cohort.^19^ Although modest compared with targeted antihypertensive pharmacotherapy, this reduction is comparable with the BP benefits of structured lifestyle interventions.^20^^,^^21^

The high-frequency monitoring also revealed a temporal lag between the immediate increase in weight monitoring and the gradual rise in BP tracking, suggesting that BP vigilance evolves as a secondary priority after weight loss.

### Comparison With Trial Evidence and Cardiovascular Outcomes

The magnitude of weight loss observed (∼10%) aligns with, although is more modest than, the efficacy reported in landmark pivotal randomized controlled trials, such as STEP 1 for semaglutide 2.4 mg (14.9%).^1^ This attenuation likely reflects the efficacy-effectiveness gap inherent to routine practice (variable adherence and unmonitored titration), compounded by our cohort comprising a composite of GLP-1 RA molecules and dosages, whereas trials typically evaluate a single high-potency agent at maximal dose.^22^^,^^23^

Although this exposure heterogeneity precludes molecule and dosage-specific comparisons, the study is designed to capture the broad effectiveness of these therapies as they were applied in our real-world setting. Our analysis represents a form of completer analysis defined by sustained device engagement rather than drug continuity^24^ and thus includes users who may have discontinued medication but continued monitoring. Regarding BP, the observed 2.5 mm Hg systolic reduction aligns with secondary endpoints from the STEP trials 1 and is associated with a tangible decrease in cardiovascular risk.^25^^,^^26^

### Weight Loss Quality and Physiological Heterogeneity

Regarding body composition, a primary concern with GLP-1 RAs is the potential for sarcopenia. Historically, lean body mass accounts for approximately 25% of weight lost during standard caloric restriction.^27^ In our cohort, the estimated muscle loss (21.6%) compared favorably with this benchmark, suggesting that the physiological quality of weight loss in this real-world setting is at least comparable with caloric restrictions. However, we acknowledge that clinical sarcopenia involves functional and strength deficits that were not assessed in this study.

Besides, our findings align with the SEMALEAN study (n=115), which observed a biphasic remodeling pattern where lean mass decline stabilized after 7 months.^6^ However, we did not observe the complete muscle sparing reported in smaller cohorts treated with oral semaglutide.^28^

Beyond aggregate trends, weight loss outcomes varied across baseline physiological profiles. Women, younger adults, and individuals with a lower MFR achieved greater relative weight reductions. Among all covariates examined, the baseline MFR was the strongest independent predictor of weight loss quality, retained in 100% of bootstrap replicates for both fat and muscle mass outcomes. Patients with an altered body composition (low MFR) lost more fat and spared more muscle, whereas those with a favorable profile (high MFR) experienced proportionally greater muscle loss relative to fat loss. This gradient suggests that the body composition response to GLP-1 RAs is conditioned by the patient’s starting physiological state. If confirmed, baseline body composition assessment could help set realistic expectations and identify patients requiring closer monitoring for muscle loss.

### Limitations

Our findings must be interpreted in light of several limitations.

1. Exposure quality: We relied on self-reported GLP-1 RA use without access to confirmed prescriptions or specific dosages. However, although this precludes molecule-specific analysis, our study captures the aggregate effectiveness of the therapeutic class because it was used in our real-life cohort.
2. Measurement limitations: We relied on proprietary BIA, which is sensitive to a person’s hydration level, and GLP-1 RAs can sometimes cause dehydration through side effects such as nausea or increased salt excretion.^3^ Because BIA uses water to estimate muscle mass, the scale may mistake fluid loss for actual muscle loss. However, this means our device-estimated muscle change likely overestimates the amount of muscle lost, making our safety conclusions conservative.
3. Diabetes information: We lacked universal access to type 2 diabetes status. While partial data from supplementary health forms suggests a low prevalence of type 2 diabetes (∼3%-4%) among participants with known status, the lack of clinical history for the entire cohort remains a limitation because metabolic status can influence the magnitude of weight loss response.
4. Unmeasured confounding: Although we adjusted for age, gender, activity, and pretreatment trends, matching cannot fully eliminate confounding by indication or concurrent lifestyle changes. Our results remain subject to unmeasured factors, such as concurrent medications, structured exercise programs, dietary shifts, or other intentional behavior modifications, not captured by the devices.
5. Selection bias: Our cohort excludes users who ceased monitoring entirely, potentially introducing informative censoring. However, in connected health ecosystems, self-monitoring tends to persist as a behavioral habit independent of active therapy.^29^ Although users may weigh themselves less frequently after discontinuing a drug, seldom stop monitoring completely.
6. Generalizability: The cohort consists of connected device users who may be more health-conscious than the general population, providing a “best-case” benchmark for efficacy and adherence.
7. Statistical power and follow-up: The BP cohort was underpowered to robustly validate independent effect modifiers. Additionally, the 1-year observation window precludes assessment of long-term durability or potential rebound effects following discontinuation.

## Conclusion And Future Directions

Despite these limitations, this study suggests that GLP-1 RA initiation in a real-world setting is associated with clinically significant weight loss driven predominantly by device-estimated fat mass reduction, alongside sustained BP improvements. Baseline body composition, measured by the MFR, was a strong predictor of weight loss quality: patients with the most altered profiles lost more fat and spared more muscle, whereas those with a favorable body composition experienced proportionally greater muscle loss. These findings suggest that accounting for baseline body composition may help clinicians anticipate individual treatment responses and identify patients who require closer monitoring—a step toward individualized precision medicine in GLP-1 RA therapy.

Future research requires larger cohorts to characterize the heterogeneity of BP response, which our study was underpowered to detect, and to confirm whether baseline MFR can serve as a reliable clinical predictor of body composition outcomes under GLP-1 RA therapy. Longer follow-up periods are also essential to assess the durability of these effects and to capture the dynamics of long-term maintenance versus discontinuation. Methodologically, a transition toward functional causal inference (treating patient trajectories as continuous functions rather than static distributions), could offer greater precision for identifying patterns of maintenance versus relapse.

## Potential Competing Interests

Ms Chabassier is a doctoral candidate supported by a CIFRE grant funded by Withings and the Association Nationale de la Recherche et de la Technologie (ANRT). Dr Vittrant and Mr Binon are full-time employees of Withings. Dr Josse and Dr Scornet declare no competing interests.

## Ethics Statement

This study was a retrospective observational cohort analysis using longitudinal data from Withings connected health devices. All participants provided digital informed consent for the use of their deidentified data for scientific research purposes via the Withings application. The study was conducted in strict adherence to the GDPR and Health Insurance Portability and Accountability Act (HIPAA) security standards to ensure the protection and privacy of all participant data.

## Declaration of Generative AI and AI-Assisted Technologies in the Writing Process

During the preparation of this work, the authors used GitHub Copilot and Gemini Advanced to assist with code optimization and to refine the linguistic clarity of the manuscript. After using this tool, the authors reviewed and edited the content as needed and takes full responsibility for the content of the publication.
