# Supplementary material for: Real-World Body Composition and Blood Pressure Changes Following Glucagon-Like Peptide 1 Receptor Agonist Initiation: A Causal Inference Study of Connected Device Data
Source: Mayo Clin Proc Digit Health. 2026 Jul 28;4(3):100375. doi: 10.1016/j.mcpdig.2026.100375 (PMC13576509; doi:10.1016/j.mcpdig.2026.100375)
Supplement: Supplementary file 1 [file mmc1.pdf]

# Real-world body composition and blood pressure changes following GLP-1 Receptor Agonist initiation: a distributional causal inference study of connected device data

## Table of Contents


**1 Supplementary methods** . . . **1**
1.1 Data privacy and security protocols . . . 1
1.2 Distributional causal inference framework (ADD-MALTS) . . . 1
1.2.1 Adaptive metric learning . . . 1
1.2.2 Distributional matching . . . 1
1.2.3 Effect estimation . . . 1
1.3 Treatment quality and imputation ($t_0$) . . . 1
1.4 Justification of the primary efficacy window . . . 2
1.5 Bootstrap-based variable selection for heterogeneity . . . 2
1.5.1 Supportive model 1: Event-study Linear Mixed-Effects Model (LMEM) . . . 2
1.5.2 Supportive model 2: Person Fixed-Effects (FE) model . . . 3
1.5.3 Key mathematical differences . . . 4
1.5.4 Hypothesis testing for supportive models . . . 4
1.6 Sensitivity analyses for distributional matching . . . 4
1.7 Technical considerations regarding regression to the mean and model specification . . . 4
1.8 Software and reproducibility . . . 5
1.9 Missing data . . . 5
1.10 Device characteristics and distribution . . . 5
1.10.1 Smart scales and measurement procedure . . . 6
1.10.2 Blood pressure monitors and measurement procedure . . . 6
1.10.3 Hardware distribution across cohorts . . . 6
1.11 Ethics . . . 7

**2 Supplementary results** . . . **8**
2.1 Sensitivity analyses for distributional matching . . . 8
2.2 Comparison with parametric models . . . 8

**3 Supplementary tables** . . . **9**

**4 Supplementary figures** . . . **14**

# 1 SUPPLEMENTARY METHODS

## 1.1 Data privacy and security protocols

To ensure participant anonymity, data presented in this manuscript were aggregated at the cohort or subgroup level (e.g., means, barycentres). Where unit-level data processing was required for analysis, records were fully pseudonymised, and a privacy-preserving perturbation mechanism (random perturbation up to ±2% followed by rounding) was applied to raw measurements to prevent re-identification. The hosting and processing infrastructure complies with HDS (Health Data Hosting), ISO 27001, ISO 27701, and HIPAA security standards.

## 1.2 Distributional causal inference framework (ADD-MALTS)

To address the complexity of high-frequency digital biomarkers and baseline physiological heterogeneity, we employed the ADD-MALTS framework. Unlike standard matching methods that compress patient history into scalar averages (obscuring volatility), this approach matches users based on their full pre-treatment outcome distribution.

### *1.2.1 Adaptive metric learning*

Using a 20% training split of the unexposed cohort, the algorithm learned a distance metric that weighted covariates based on their prognostic importance. This integrated static confounders (age, gender, BMI, baseline steps) with the full pre-inclusion outcome distribution, quantified via Wasserstein distance.

### *1.2.2 Distributional matching*

On the estimation set (80%), following ADD-MALTS, we performed nearest-neighbour matching ($k = 5$). For each user, a synthetic counterfactual distribution was generated as the Wasserstein barycentre of their matched neighbours.

### *1.2.3 Effect estimation*

The distributional Average Treatment Effect on the Treated (dATT) was estimated as the quantile-wise difference between the Wasserstein barycentre of the observed exposed outcome distributions and the Wasserstein barycentre of the estimated counterfactual distributions during the efficacy window. We derived the scalar Average Treatment Effect on the Treated (sATT) by computing the difference between the medians ($\tau_{0.5}$) of these two aggregate distributions.

## 1.3 Treatment quality and imputation ($t_0$)

Our analysis was based on self-reported GLP-1 RA use and lacked access to confirmed prescriptions, specific dosages, or adherence logs. Although this prevented molecule-specific comparisons, the study characterizes the aggregate outcomes of the therapeutic class within a routine clinical environment. Furthermore, the distinct and immediate drop in weight trajectories observed at the imputed initiation date ($t_0$) provides reassuring evidence regarding the validity of our start-date imputation process.

To approximate the start of therapy ($t_0$) from categorical survey responses, we imputed a specific index date using the rounded midpoint of the reported duration interval. The mapping logic was as follows:

- "Less than 3 month": $t_0$ = date of survey - 1.5 months (rounded to 2 months)
- "3–6 months": $t_0$ = date of survey - 4.5 months (rounded to 5 months)

– "6–12 months": $t_0$ = date of survey - 9 months

Users reporting usage greater than one year were excluded to minimize the risk of temporal misclassification error.

### 1.4 Justification of the primary efficacy window

The selection of the 10–14 month window as the interval for reconstructing primary outcome distributions was guided by a balance of clinical, statistical, and longitudinal requirements:

1. **Results interpretability:** We prioritized an endpoint **centered at 12 months** to enhance the clarity of the findings (e.g., to allow for meaningful comparisons with the one-year outcomes reported in landmark GLP-1 RA clinical trials).
2. **Distributional density:** To reconstruct high-resolution outcome distributions, the window required a sufficient density of data points. While the body composition cohort showed a median of 7–10 measurements per month, the blood pressure cohort recorded a median of 2–3. A window narrower than four months would have provided insufficient data to reliably characterize the measurement distributions for the blood pressure analysis.
3. **Temporal precision:** Conversely, a wider window (e.g., 6 months) was deemed insufficiently precise given the rapid velocity of physiological changes observed following GLP-1 initiation. Indeed, a broad window risks masking the specific 12-month state by averaging together different stages of the weight loss trajectory.
4. **Cohort retention:** This boundary aligns with our longitudinal retention requirement. As demonstrated in the cumulative distribution of last measurement times (figure S15), month 14 represents the latest boundary where over 90% of the initiators remain active for assessment. This ensures that the primary efficacy analysis is representative of the initiated population rather than a small subset of long-term users.

### 1.5 Bootstrap-based variable selection for heterogeneity

In addition to computing subgroups effects (sCATT) by age, self-reported gender identity, and baseline BMI, we employed a bootstrap-based best linear projection procedure to identify the main drivers of heterogeneity of treatment effects among these correlated variables.

Within each of the 500 bootstrap replicates, we modelled the scalar unit-level treatment effect using multivariable linear regression initialised with all baseline characteristics (age, gender, initial BMI, baseline steps, baseline BP category for Cohort 2). We then applied a backward stepwise selection (minimising AIC) to identify the optimal predictor subset. A variable was classified as a robust effect modifier only if it was retained in the model in >70% of bootstrap replicates. For each variable, we report the median value of the coefficients $\beta$ computed across bootstraps and the corresponding 95% confidence interval to quantify the direction and magnitude of the heterogeneity.

#### *1.5.1 Supportive model 1: Event-study Linear Mixed-Effects Model (LMEM)*

This model estimates the average effect of treatment while accounting for correlation between repeated measurements on the same person using a random intercept.

The model is specified as:

$$
\begin{aligned}
\text{value}_{it} \;=\;& \overbrace{\alpha_i}^{\text{Random intercept}} \quad + \beta_1 \cdot \text{baseline\_value}_i \;+\; \overbrace{\sum_k \beta_{2,k} \cdot \text{Covariate}_{ik}}^{\text{Static covariates (age, gender, BMI, etc.)}} \\
&+ \overbrace{\sum_j \gamma_j \cdot \text{Time}_{tj}}^{\text{Time dummies}} \;+\; \overbrace{\sum_l \theta_l \cdot \text{Month}_{tl}}^{\text{Seasonality (month of year)}} \\
&+ \overbrace{\sum_j \delta_j \cdot (\text{Time}_{tj} \times \text{treatment}_i)}^{\text{Event-study (DiD) terms}} + \varepsilon_{it}
\end{aligned}
$$

Where:

- $\text{value}_{it}$ is the outcome for person $i$ at relative time $t$.
- $\alpha_i$ is the random intercept for person $i$, assumed to be normally distributed: $\alpha_i \sim \mathcal{N}(\mu, \sigma_\alpha^2)$. This term captures unobserved, stable heterogeneity between individuals.
- $\text{baseline\_value}_i$ is the scalar baseline outcome value for person $i$.
- $\text{Covariate}_{ik}$ are the $k$ static (time-invariant) covariates for person $i$, such as age, gender, and baseline BMI.
- $\text{Time}_{tj}$ is a binary indicator (dummy variable) for the $j$-th relative time period, with month -1 as the reference category.
- $\text{Month}_{tl}$ is a binary indicator for the $l$-th calendar month (1-12) to control for seasonality.
- $\text{treatment}_i$ is a binary indicator, equal to 1 if person $i$ is in the treated group and 0 otherwise.
- $\delta_j$ is the parameter of interest. It represents the ATT at time period $j$ relative to the reference period (month -1).
- $\varepsilon_{it}$ is the random error term.

The model parameters are estimated using Restricted Maximum Likelihood (REML).

### *1.5.2 Supportive model 2: Person Fixed-Effects (FE) model*

This model provides a more stringent robustness check by removing all time-invariant confounding. The model is specified as:

$$
\begin{aligned}
\text{value}_{it} \;=\;& \overbrace{\alpha_i}^{\text{Person fixed effect}} \quad + \overbrace{\sum_j \gamma_j \cdot \text{Time}_{tj}}^{\text{Time dummies}} \;+\; \overbrace{\sum_l \theta_l \cdot \text{Month}_{tl}}^{\text{Seasonality (month of year)}} \\
&+ \overbrace{\sum_j \delta_j \cdot (\text{Time}_{tj} \times \text{treatment}_i)}^{\text{Event-study (DiD) terms}} + \varepsilon_{it}
\end{aligned}
$$

Where:

- $\text{value}_{it}$, $\text{Time}_{tj}$, $\text{Month}_{tl}$, $\text{treatment}_i$, $\delta_j$, and $\varepsilon_{it}$ are defined as in the LMEM.
- $\alpha_i$ is the person fixed effect. This is a unique, fixed parameter for each person $i$, representing all unobserved, time-invariant characteristics of that individual (e.g., genetics, geography, long-term motivation).

This model is estimated via OLS (Ordinary Least Squares) after demeaning all variables within each person (the "within transformation"). Standard errors are clustered by person to account for serial correlation in the error term $\varepsilon_{it}$.

#### 1.5.3 Key mathematical differences

- Intercepts ($\alpha_i$): The LMEM assumes the intercepts $\alpha_i$ are random variables drawn from a single population distribution. The FE model treats each $\alpha_i$ as a fixed parameter to be estimated (or "absorbed"), making no assumption about their distribution.
- Time-invariant covariates: The LMEM explicitly includes time-invariant covariates like baseline_value, age, and gender. The FE model cannot estimate the effect of these covariates, as they are perfectly collinear with the person fixed-effect $\alpha_i$ and are removed by the demeaning process. The FE model's key strength is that it controls for all such time-invariant covariates, even those that are unmeasured.

#### 1.5.4 Hypothesis testing for supportive models

For the two supportive event-study models (LMEM and FE), we prespecified two formal hypotheses. These tests were based on the vector of event-study coefficients ($\delta_j$ in our model equations), where each $\delta_j$ represents the estimated ATT for a specific month $j$ relative to the inclusion date.

- Primary hypothesis: Our primary hypothesis tested the average ATT during the pre-specified primary endpoint window (months 10 to 14 post-inclusion). This was estimated as a linear contrast of the relevant post-treatment coefficients, testing the null hypothesis that their average was zero.

$$H_0 : \frac{1}{5}\sum_{j=10}^{14} \delta_j = 0$$

- Secondary hypothesis: Our secondary hypothesis tested the average ATT over the entire 14-month follow-up period (months 0 to 14). This was also estimated as a linear contrast of the corresponding event-study coefficients, testing the null hypothesis that their average was zero.

$$H_0 : \frac{1}{15}\sum_{j=0}^{14} \delta_j = 0$$

### 1.6 Sensitivity analyses for distributional matching

To assess the robustness of the primary ADD-MALTS framework, we conducted two specific sensitivity checks:

- Number of neighbours: The entire pipeline was re-executed varying the number of nearest neighbours ($k$) from the primary value ($k = 5$) to stricter ($k = 2$) and looser ($k = 10$) specifications.
- Temporal uncertainty: To address potential misclassification in the self-reported start date, we re-imputed $t_0$ by shifting the index date to the lower and upper boundaries of the reported duration intervals (effectively shifting the start date by $\pm$ 2–3 months depending on the interval width) and re-estimated the dATT.

### 1.7 Technical considerations regarding regression to the mean and model specification

The use of a matched counterfactual group in this study was designed to address several challenges inherent in observational health data, specifically regression to the mean and functional mis-specification.

*Adjustment for regression to the mean*

In obesity research, treatment initiation often coincides with a period of peak Body Mass Index (BMI). These baseline measurements may represent a transient maximum that tends to naturally decline toward a long-term average over time, regardless of intervention. A naive “pre-post” analysis—which compares a user only to their own baseline—would likely attribute this natural drift to the therapy, thereby overestimating the treatment effect.

By matching exposed users to controls with similar pre-treatment trajectories and high baseline BMIs, the framework identifies unexposed individuals in a similar transient physiological state. The observed reduction in outcomes within the matched control group (figures 2 and 4) provides an empirical estimate of this natural physiological drift. Incorporating this counterfactual allows for the differentiation between drug-associated changes and statistical regression to the mean.

*Non-parametric distributional modeling*

The choice of the ADD-MALTS framework was further motivated by the high-frequency nature of connected device data. While standard parametric models (such as the LMEM and FE models used in our sensitivity analyses) provide useful benchmarks, they rely on linear assumptions that may not fully capture complex, non-linear physiological trajectories.

The non-parametric approach offers two specific methodological attributes:

1. It ensures statistical consistency without requiring the specification of a rigid functional form for weight loss or blood pressure dynamics.
2. By matching on full six-month distributions rather than scalar averages, the model explicitly accounts for physiological volatility and measurement frequency, which are often obscured when longitudinal history is compressed into a single mean value.

### 1.8 Software and reproducibility

Statistical analyses and data processing were conducted using Python (version 3.10.4). Data manipulation and numerical computations were performed using Pandas, NumPy and Scikit-learn. The ADD-MALTS implementation relies on standard scientific computing libraries, primarily NumPy and Pandas for data structures, SciPy for statistical optimization and z-score calculations, and Scikit-learn for the linear and ensemble models used in the matching and estimation phases. Supportive parametric modeling, including linear mixed-effects and fixed-effects specifications, was implemented using Statsmodels and SciPy. Data visualisations, including distributional plots and longitudinal trajectories, were generated using Matplotlib and Seaborn. Project management and version control were maintained using GitLab (version 18.7).

### 1.9 Missing data

Missingness for the baseline step count covariate was negligible (2.5% in Cohort 1; 2.8% in Cohort 2) and was imputed by the mean.

### 1.10 Device characteristics and distribution

The data used in this study were generated by Withings connected health devices. Measurements are initiated spontaneously by the user without digital prompts, incentives, or reminders from the Withings application. Body composition

data were collected via smart scales using Bioelectrical Impedance Analysis (BIA), and blood pressure data were collected via oscillometric blood pressure monitors (BPM).

#### *1.10.1 Smart scales and measurement procedure*

Measurements are initiated spontaneously by the user simply stepping onto the scale. The devices automatically recognize the specific user based on weight and body composition history. In cases where users have similar profiles, the scale prompts the user to select the correct identity via a weight-shift interface (leaning in a specific direction), or the measurement can be manually assigned in the Withings application.

Critically, all scale models included in this study share a standardized hardware architecture for the primary outcomes of weight and body composition. Total weight is measured via high-precision load cells, while fat and muscle mass are estimated using a common multi-electrode BIA platform. This ensures that the primary longitudinal metrics—total fat mass and total muscle mass—are technically consistent and comparable across the device models. The devices send a low-level, imperceptible electrical current through the feet to measure tissue impedance. This impedance, combined with the user's height, weight, age, and self-reported gender, is used to estimate body compartments. All devices are Wi-Fi and Bluetooth enabled, ensuring data are automatically synchronized to the user's profile immediately following the measurement.

- **Body Plus / Body Smart:** These models use the standardized BIA platform to provide the primary estimates of muscle mass and fat mass used in this study. While the Body Smart model includes additional frequencies for water compartment differentiation, the aggregate muscle mass calculation remains consistent with the other models.
- **Body Cardio / Body Comp:** These models incorporate the same core BIA technology for body composition while including additional sensors for cardiovascular or metabolic markers. These auxiliary features do not alter the underlying methodology for weight or mass estimation.
- **Body Scan:** This station uses the core BIA architecture but adds a retractable handle for segmental analysis. While this allows for localized measurement, the primary metrics for total body fat and total muscle mass used in our analysis are derived from the same standardized algorithms as the other models.

#### *1.10.2 Blood pressure monitors and measurement procedure*

Blood pressure is measured using a portable cuff placed around the upper arm. The user starts the process by pressing a single button on the device. For devices shared by multiple users, the correct profile is selected directly on the monitor by using a long-press on the button to toggle through names displayed on the LED matrix. Once the measurement is complete, data are synchronized automatically via Wi-Fi or Bluetooth to the user's digital profile. The majority of BP data were captured by the **BPM Connect**, a Wi-Fi-enabled oscillometric monitor. This device has been clinically validated to meet the requirements of the AAMI/ESH/ISO Universal Standard, ensuring its accuracy in the general population.

#### *1.10.3 Hardware distribution across cohorts*

To ensure findings were not biased by specific hardware versions, we assessed the distribution of device models across groups. The distribution remained consistent between the exposed and matched control groups, indicating that the observed associations are not artifacts of specific device types.

**Table: Device model distribution by study group (After adjustment)**

| Scale Models (Cohort 1) | Treated (%) | Control (%) |
|---|---|---|
| Body Cardio | 17.1% | 17.7% |
| Body Comp | 12.9% | 14.3% |
| Body Plus | 47.1% | 40.3% |
| Body Scan | 12.3% | 13.8% |
| Body Smart | 10.6% | 13.9% |
| **Total** | **100.0%** | **100.0%** |

| BPM Models (Cohort 2) | Treated (%) | Control (%) |
|---|---|---|
| BPM (Classic/Wired) | 5.9% | 4.4% |
| BPM Connect | 92.1% | 93.3% |
| BPM Vision | 2.0% | 2.3% |
| **Total** | **100.0%** | **100.0%** |

### 1.11 Ethics

In addition to GDPR compliance, this study adhered to the specific recommendations of the French Data Protection Authority (Commission Nationale de l'Informatique et des Libertés; CNIL).

## 2 SUPPLEMENTARY RESULTS

### 2.1 Sensitivity analyses for distributional matching

To assess the stability of the ADD-MALTS estimates, we conducted sensitivity checks varying the matching parameters and temporal definitions.

- Matching stringency: Varying the number of nearest neighbours from the primary specification ($k = 5$) to stricter ($k = 2$) or looser ($k = 10$) definitions did not materially alter the median dATT estimates for body weight (range: –9.7% to –9.9%) or systolic blood pressure (range: –2.5 to –2.8 mm Hg).
- Temporal uncertainty: Re-imputing the index date ($t_0$) by shifting the start date to the lower and upper boundaries of the reported duration intervals ($\pm$ 2 months) yielded consistent trajectories, confirming that the findings are not artifacts of the mid-point imputation strategy.

### 2.2 Comparison with parametric models

Two alternative specifications were estimated to validate the primary distributional findings (Table S2).

- Linear Mixed-Effects Model (LMEM): Estimated an ATT of –11.7% [95% CI –11.8, –11.5] for body weight and –3.7 mm Hg [–4.6, –2.7] for systolic BP.
- User-Fixed Effects (FE): Estimated an ATT of –12.8% [–14.1, –11.5] for body weight and –4.8 mm Hg [–7.2, –2.4] for systolic BP.

While the magnitude of the effect was slightly larger in the parametric models (likely due to the linearity assumptions and different handling of time-invariant confounding), the direction and statistical significance of all outcomes were fully consistent with the primary distributional analysis.

## 3 SUPPLEMENTARY TABLES

Table S1 **Baseline characteristics of the unadjusted and adjusted (estimation) cohorts.** . . . . . . . . 10

Table S2 **Estimated effect of GLP-1 RA initiation on body composition and blood pressure using supportive linear event-study models.** . . . . . . . . . . . . . . . . . . . . . . . . . . . . . . . . 12

Table S3 **Estimated effect of GLP-1 RA initiation over the complete follow-up period (months 0–14) using supportive linear event-study models.** . . . . . . . . . . . . . . . . . . . . . . . . . . . . 13

## Table S1: Baseline characteristics of the unadjusted and adjusted (estimation) cohorts.

**Cohort 1: Body Composition Analysis**

| | UNADJUSTED (FULL DATA) | | | ADJUSTED (Weight %) | | | ADJUSTED (Muscle %) | | | ADJUSTED (Fat %) | | |
|---|---|---|---|---|---|---|---|---|---|---|---|---|
| **Characteristic** | **With** | **Without** | **Overall** | **With** | **Without** | **Overall** | **With** | **Without** | **Overall** | **With** | **Without** | **Overall** |
| **Total N** | 396 | 1635 | 2031 | 324 [320, 329] | 1339 [1334, 1344] | 1663 [1660, 1667] | 324 [319, 329] | 1339 [1333, 1344] | 1663 [1659, 1667] | 325 [320, 330] | 1339 [1332, 1345] | 1663 [1660, 1667] |
| **Age 20-39** | 58 (14.6%) | 359 (22.0%) | 417 (20.5%) | 48 (14.7%) | 294 (22.0%) | 342 (20.5%) | 47 (14.6%) | 294 (21.9%) | 341 (20.5%) | 47 (14.6%) | 294 (22.0%) | 342 (20.5%) |
| **Age 40-59** | 250 (63.1%) | 950 (58.1%) | 1200 (59.1%) | 205 (63.1%) | 778 (58.1%) | 983 (59.1%) | 205 (63.2%) | 778 (58.1%) | 983 (59.1%) | 205 (63.1%) | 778 (58.1%) | 982 (59.1%) |
| **Age 60+** | 88 (22.2%) | 326 (19.9%) | 414 (20.4%) | 72 (22.2%) | 267 (19.9%) | 339 (20.4%) | 72 (22.2%) | 267 (19.9%) | 339 (20.4%) | 72 (22.3%) | 267 (19.9%) | 339 (20.4%) |
| **Missing** | 0 (0.0%) | 0 (0.0%) | 0 (0.0%) | 0 (0.0%) | 0 (0.0%) | 0 (0.0%) | 0 (0.0%) | 0 (0.0%) | 0 (0.0%) | 0 (0.0%) | 0 (0.0%) | 0 (0.0%) |
| **Underweight** | 1 (0.3%) | 9 (0.6%) | 10 (0.5%) | 15 (4.5%) | 318 (23.8%) | 333 (20.0%) | 15 (4.5%) | 318 (23.8%) | 333 (20.0%) | 15 (4.5%) | 318 (23.7%) | 332 (20.0%) |
| **Healthy** | 17 (4.3%) | 379 (23.2%) | 396 (19.5%) | 85 (26.2%) | 522 (39.0%) | 607 (36.5%) | 85 (26.2%) | 522 (39.0%) | 607 (36.5%) | 86 (26.4%) | 522 (39.0%) | 608 (36.5%) |
| **Overweight** | 104 (26.3%) | 638 (39.0%) | 742 (36.5%) | 95 (29.4%) | 304 (22.7%) | 399 (24.0%) | 95 (29.3%) | 304 (22.7%) | 399 (24.0%) | 95 (29.3%) | 304 (22.7%) | 399 (24.0%) |
| **Obesity** | 274 (69.2%) | 609 (37.2%) | 883 (43.5%) | 129 (39.9%) | 195 (14.6%) | 324 (19.5%) | 129 (39.9%) | 195 (14.5%) | 324 (19.5%) | 129 (39.8%) | 195 (14.6%) | 324 (19.5%) |
| **Missing** | 0 (0.0%) | 0 (0.0%) | 0 (0.0%) | 0 (0.0%) | 0 (0.0%) | 0 (0.0%) | 0 (0.0%) | 0 (0.0%) | 0 (0.0%) | 0 (0.0%) | 0 (0.0%) | 0 (0.0%) |
| **Female** | 180 (45.5%) | 670 (41.0%) | 850 (41.9%) | 147 (45.3%) | 549 (41.0%) | 696 (41.8%) | 148 (45.5%) | 549 (41.0%) | 697 (41.9%) | 147 (45.3%) | 549 (41.0%) | 696 (41.8%) |
| **Male** | 216 (54.5%) | 965 (59.0%) | 1181 (58.1%) | 177 (54.7%) | 790 (59.0%) | 967 (58.2%) | 177 (54.5%) | 789 (59.0%) | 966 (58.1%) | 178 (54.7%) | 790 (59.0%) | 967 (58.2%) |
| **Missing** | 0 (0.0%) | 0 (0.0%) | 0 (0.0%) | 0 (0.0%) | 0 (0.0%) | 0 (0.0%) | 0 (0.0%) | 0 (0.0%) | 0 (0.0%) | 0 (0.0%) | 0 (0.0%) | 0 (0.0%) |
| **Inactive** | 158 (39.9%) | 428 (26.2%) | 586 (28.9%) | 130 (39.9%) | 350 (26.2%) | 480 (28.8%) | 130 (40.0%) | 350 (26.2%) | 480 (28.9%) | 130 (39.9%) | 350 (26.2%) | 480 (28.8%) |
| **Mod. Act.** | 196 (49.5%) | 936 (57.2%) | 1132 (55.7%) | 161 (49.5%) | 767 (57.3%) | 927 (55.7%) | 160 (49.4%) | 767 (57.3%) | 927 (55.7%) | 161 (49.5%) | 766 (57.3%) | 927 (55.7%) |
| **Active** | 22 (5.6%) | 241 (14.7%) | 263 (12.9%) | 18 (5.5%) | 198 (14.8%) | 215 (13.0%) | 18 (5.6%) | 197 (14.7%) | 215 (12.9%) | 18 (5.6%) | 197 (14.7%) | 216 (13.0%) |
| **PA Miss.** | 20 (5.1%) | 30 (1.8%) | 50 (2.5%) | 16 (5.0%) | 25 (1.8%) | 41 (2.5%) | 16 (5.0%) | 25 (1.8%) | 41 (2.5%) | 16 (5.0%) | 25 (1.8%) | 41 (2.5%) |

**Cohort 2: Blood Pressure Analysis**

| | UNADJUSTED (FULL DATA) | | | ADJUSTED (Systolic BP) | | | ADJUSTED (Diastolic BP) | | |
|---|---|---|---|---|---|---|---|---|---|
| **Characteristic** | **With** | **Without** | **Overall** | **With** | **Without** | **Overall** | **With** | **Without** | **Overall** |
| **Total N** | 148 | 524 | 672 | 121 [119, 124] | 429 [426, 433] | 551 [549, 552] | 121 [119, 124] | 429 [426, 433] | 551 [548, 553] |
| **Age 20-39** | 11 (7.4%) | 56 (10.7%) | 67 (10.0%) | 9 (7.5%) | 46 (10.7%) | 55 (10.0%) | 9 (7.4%) | 46 (10.7%) | 55 (10.0%) |
| **Age 40-59** | 95 (64.2%) | 276 (52.7%) | 371 (55.2%) | 78 (64.1%) | 226 (52.7%) | 304 (55.2%) | 78 (64.1%) | 226 (52.7%) | 304 (55.2%) |
| **Age 60+** | 42 (28.4%) | 192 (36.6%) | 234 (34.8%) | 34 (28.4%) | 157 (36.6%) | 191 (34.8%) | 35 (28.5%) | 157 (36.6%) | 192 (34.8%) |
| **Missing** | 0 (0.0%) | 0 (0.0%) | 0 (0.0%) | 0 (0.0%) | 0 (0.0%) | 0 (0.0%) | 0 (0.0%) | 0 (0.0%) | 0 (0.0%) |
| **Underweight** | 0 (0.0%) | 5 (1.0%) | 5 (0.7%) | 3 (2.7%) | 97 (22.5%) | 100 (18.1%) | 3 (2.7%) | 96 (22.5%) | 100 (18.1%) |
| **Healthy** | 4 (2.7%) | 113 (21.6%) | 117 (17.4%) | 25 (20.2%) | 173 (40.2%) | 197 (35.8%) | 24 (20.1%) | 173 (40.3%) | 197 (35.8%) |
| **Overweight** | 30 (20.3%) | 211 (40.3%) | 241 (35.9%) | 43 (35.2%) | 107 (24.9%) | 149 (27.1%) | 43 (35.2%) | 106 (24.8%) | 149 (27.1%) |
| **Obesity** | 114 (77.0%) | 195 (37.2%) | 309 (46.0%) | 51 (41.9%) | 53 (12.4%) | 104 (18.9%) | 51 (42.0%) | 53 (12.4%) | 104 (18.9%) |
| **Missing** | 0 (0.0%) | 0 (0.0%) | 0 (0.0%) | 0 (0.0%) | 0 (0.0%) | 0 (0.0%) | 0 (0.0%) | 0 (0.0%) | 0 (0.0%) |
| **Female** | 46 (31.1%) | 127 (24.2%) | 173 (25.7%) | 84 (68.9%) | 325 (75.7%) | 409 (74.2%) | 84 (69.1%) | 325 (75.8%) | 409 (74.3%) |
| **Male** | 102 (68.9%) | 397 (75.8%) | 499 (74.3%) | 38 (31.1%) | 104 (24.3%) | 142 (25.8%) | 37 (30.9%) | 104 (24.2%) | 142 (25.7%) |
| **Missing** | 0 (0.0%) | 0 (0.0%) | 0 (0.0%) | 0 (0.0%) | 0 (0.0%) | 0 (0.0%) | 0 (0.0%) | 0 (0.0%) | 0 (0.0%) |
| **Inactive** | 53 (35.8%) | 146 (27.9%) | 199 (29.6%) | 43 (35.7%) | 120 (27.9%) | 163 (29.6%) | 44 (35.8%) | 120 (27.9%) | 163 (29.6%) |
| **Mod. Act.** | 80 (54.1%) | 284 (54.2%) | 364 (54.2%) | 66 (54.1%) | 232 (54.2%) | 298 (54.1%) | 66 (54.0%) | 233 (54.2%) | 298 (54.2%) |
| **Active** | 7 (4.7%) | 83 (15.8%) | 90 (13.4%) | 6 (4.7%) | 68 (15.8%) | 74 (13.4%) | 6 (4.8%) | 68 (15.8%) | 74 (13.4%) |
| **PA Miss.** | 8 (5.4%) | 11 (2.1%) | 19 (2.8%) | 7 (5.4%) | 9 (2.1%) | 16 (2.8%) | 7 (5.4%) | 9 (2.1%) | 16 (2.8%) |

*Legend and Notes for Table S1*

This table compares the demographic and physiological profiles of the full study population (**Unadjusted**) against the specific estimation sets used for the primary **ADD-MALTS** analysis (**Adjusted**). Data are reported separately for Cohort 1 (Body Composition) and Cohort 2 (Blood Pressure).

**Statistical Presentation:**

- **Unadjusted Columns:** Values represent the observed frequency ($N$) and percentage (%) of the full cohorts prior to matching.
- **Adjusted Columns:** Values are derived from the distribution across 500 bootstrap replicates:
  - **Total Group Sizes ($N$):** Presented as the mean count across replicates with the [1st quartile, 3rd quartile] in brackets.
  - **Subgroup Characteristics:** Presented as the mean count ($N$, rounded to the nearest integer) and mean percentage (%) across replicates.

***Note on Sample Size Variation:*** *Cohort sizes in the adjusted columns vary across bootstrap replicates because the ADD-MALTS metric learning phase employs a 20% training set sampled with replacement. Adjusted computations are performed on the estimation set comprising the remaining unique users; consequently, the size of this set fluctuates depending on the number of duplicate records drawn during the training step.*

**Abbreviations:**

**ADD-MALTS**: Adaptive Distance Distributional Matching After Learning to Stretch

**Adjusted (Weight / Muscle / Fat %)**: The outcomes of the body composition analysis, *ie* percentage change in body weight, muscle mass, and fat mass, respectively.

**Age Miss. / Missing**: Missing data for age or physiological baseline characteristics

**BMI**: Body-Mass Index

**BP**: Blood Pressure

**Mod. Act.**: Moderately Active physical activity levels (defined as 5,000–9,999 steps per day)

**PA / PA Miss.**: Physical Activity / Missing data for baseline physical activity

**Q1**: First Quartile (25th percentile)

**Q3**: Third Quartile (75th percentile)

**Table S2: Estimated effect of GLP-1 RA initiation on body composition and blood pressure using supportive linear event-study models.**

| Outcome | Model Specification | ATT Estimate [95% CI][(1)] | p-value |
|---|---|---|---|
| **COHORT 1 (BC)** | | | |
| Percentage change in weight (%) | Primary (Mixed-Effects) | -11.65 [-11.81, -11.49] | <1.0E-14 |
| | Supportive (Fixed-Effects) | -12.77 [-14.05, -11.48] | <1.0E-14 |
| Percentage Change in Muscle Mass (%) | Primary (Mixed-Effects) | -2.95 [-3.05, -2.84] | <1.0E-14 |
| | Supportive (Fixed-Effects) | -2.86 [-3.30, -2.42] | <1.0E-14 |
| Percentage Change in Fat Mass (%) | Primary (Mixed-Effects) | -8.51 [-8.66, -8.36] | <1.0E-14 |
| | Supportive (Fixed-Effects) | -9.76 [-10.76, -8.75] | <1.0E-14 |
| **COHORT 2 (BP)** | | | |
| Systolic Blood Pressure (mmHg) | Primary (Mixed-Effects) | -3.67 [-4.59, -2.74] | 9.99E-15 |
| | Supportive (Fixed-Effects) | -4.84 [-7.24, -2.43] | 8.08E-05 |
| Diastolic Blood Pressure (mmHg) | Primary (Mixed-Effects) | -1.01 [-1.62, -0.39] | 0.00126 |
| | Supportive (Fixed-Effects) | -2.15 [-3.78, -0.53] | 0.0095 |

(1) The ATT is estimated over the months 10 to 14 after inclusion.

Estimates represent the Average Treatment Effect on the Treated (ATT) during the one-year efficacy window (months 10–14 post-initiation) derived from two alternative specifications: a Linear Mixed-Effects Model (LMEM) adjusting for static covariates and seasonality, and a User-Fixed Effects (FE) model controlling for time-invariant unmeasured confounding.

**Abbreviations:**

**ATT**: Average Treatment Effect on the Treated

**BC**: Body Composition

**BP**: Blood Pressure

**CI**: Confidence Interval

**FE**: Fixed-Effects

**GLP-1 RA**: Glucagon-Like Peptide-1 Receptor Agonist

**LMEM**: Linear Mixed-Effects Model

**Table S3: Estimated effect of GLP-1 RA initiation over the complete follow-up period (months 0–14) using supportive linear event-study models.**

| Outcome | Model Specification | ATT Estimate [95% CI][1] | p-value |
|---|---|---|---|
| **COHORT 1 (BC)** | | | |
| Weight Difference (%) | Primary (Mixed-Effects) | -7.95 [-8.10, -7.79] | <1.0E-14 |
| | Supportive (Fixed-Effects) | -8.55 [-9.46, -7.63] | <1.0E-14 |
| Muscle Mass Difference (%) | Primary (Mixed-Effects) | -2.05 [-2.16, -1.95] | <1.0E-14 |
| | Supportive (Fixed-Effects) | -1.95 [-2.28, -1.62] | <1.0E-14 |
| Fat Mass Difference (%) | Primary (Mixed-Effects) | -5.77 [-5.92, -5.63] | <1.0E-14 |
| | Supportive (Fixed-Effects) | -6.51 [-7.23, -5.78] | <1.0E-14 |
| **COHORT 2 (BP)** | | | |
| Systolic Blood Pressure (mmHg) | Primary (Mixed-Effects) | -3.33 [-4.22, -2.44] | 2.5E-13 |
| | Supportive (Fixed-Effects) | -4.42 [-6.24, -2.59] | 2.1E-06 |
| Diastolic Blood Pressure (mmHg) | Primary (Mixed-Effects) | -0.58 [-1.17, 0.01] | 5.2E-02 |
| | Supportive (Fixed-Effects) | -1.69 [-2.75, -0.62] | 1.9E-03 |

(1) The ATT is estimated over the months 0 to 14 after inclusion.

Estimates represent the Average Treatment Effect on the Treated (ATT) averaged over the entire post-inclusion period (months 0 to 14), rather than the specific 1-year efficacy window reported in the main text. Results are derived from two alternative specifications: a Linear Mixed-Effects Model (LMEM) adjusting for static covariates and seasonality, and a User-Fixed Effects (FE) model controlling for time-invariant unmeasured confounding.

**Abbreviations:**

**ATT**: Average Treatment Effect on the Treated

**BC**: Body Composition

**BP**: Blood Pressure

**CI**: Confidence Interval

**FE**: Fixed-Effects

**GLP-1 RA**: Glucagon-Like Peptide-1 Receptor Agonist

**LMEM**: Linear Mixed-Effects Model

## 4 SUPPLEMENTARY FIGURES


Figure S1 **Assessment of covariate balance before and after distributional matching.** . . . 15

Figure S2 **Heterogeneity of treatment effect on BP by baseline characteristics.** . . . 16

Figure S3 **Temporal density of measurements for exposed and unexposed users.** . . . 17

Figure S4 **Distribution of baseline characteristics prior to matching.** . . . 18

Figure S5 **Absolute distributional and longitudinal effects of GLP-1 RA initiation on body composition (kg).** . . . 19

Figure S6 **Comparison of smoothed versus raw longitudinal trajectories.** . . . 20

Figure S7 **Heterogeneity of treatment effect on absolute weight and body composition changes (kg).** . . . 21

Figure S8 **Seasonal distribution of inclusion dates ($t_0$).** . . . 22

Figure S9 **Comparison of smoothed versus raw longitudinal blood pressure trajectories.** . . . 23

Figure S10 **Heterogeneity of treatment effect on weight loss (%) by baseline characteristics.** . . . 24

Figure S11 **Heterogeneity of treatment effect on device-estimated muscle mass loss (%) by baseline characteristics.** . . . 25

Figure S12 **Heterogeneity of treatment effect on device-estimated fat mass loss (%) by baseline characteristics.** . . . 26

Figure S13 **Heterogeneity of treatment effect on systolic blood pressure by baseline characteristics.** . . . 27

Figure S14 **Heterogeneity of treatment effect on diastolic blood pressure by baseline characteristics.** . . . 28

Figure S15 **Cumulative distribution of last measurement time since index date ($t_0$) for Cohort 1** . . . 29

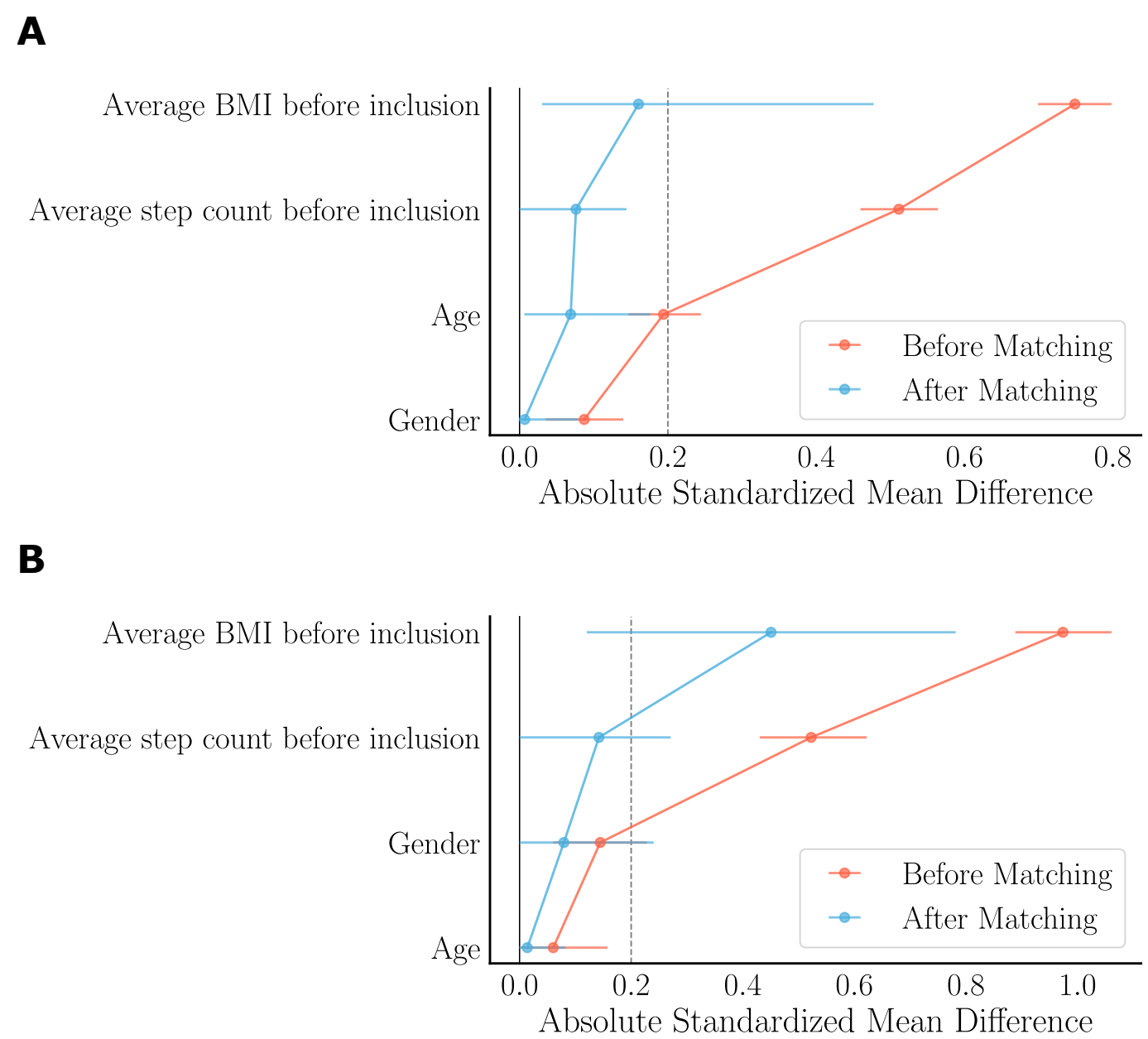


**Figure S1: Assessment of covariate balance before and after distributional matching.** Absolute Standardised Mean Differences (ASMDs) for baseline covariates in Cohort 1 (body composition) and Cohort 2 (blood pressure). The plot compares the balance between exposed and unexposed users before matching (red) and after applying the ADD-MALTS distributional matching process (blue). The vertical dashed line at 0.2 represents the pre-specified threshold for acceptable balance. Points represent the mean ASMD across 500 bootstrap replicates, and error bars indicate the estimated 95% CI. A reduction in ASMD indicates improved similarity between the exposed group and the constructed counterfactual group.
*Abbreviations: ASMD, Absolute Standardised Mean Difference; CI, Confidence Interval.*

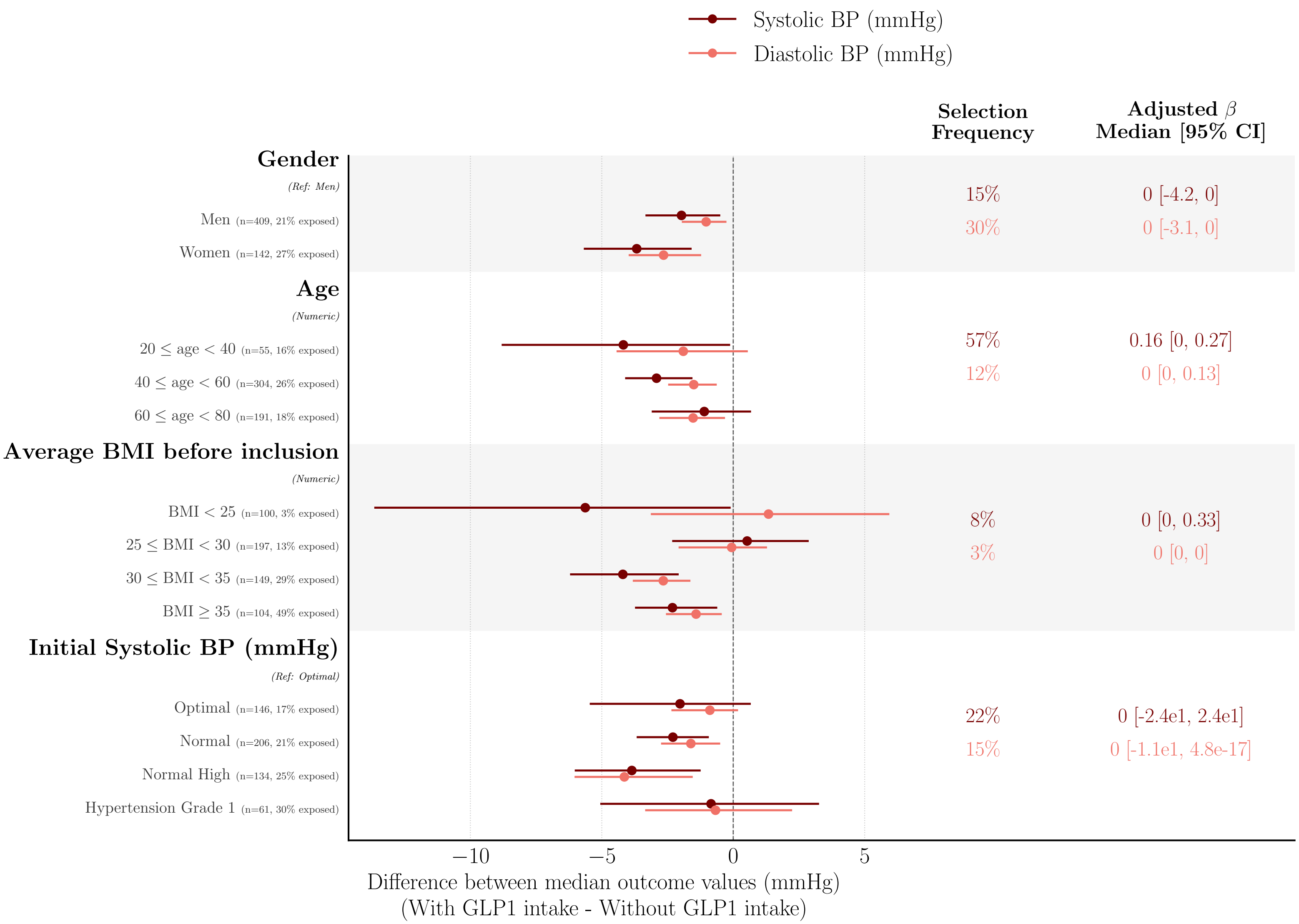


**Figure S2: Heterogeneity of treatment effect on BP by baseline characteristics.** Forest plot displaying the scalar Conditional Average Treatment Effect on the Treated (sCATT) on systolic and diastolic blood pressure across stratifications of self-reported gender identity, age, baseline BMI and systolic blood pressure category in the 6 months before $t_0$. Points represent the median difference between the barycentre distributions of the exposed and unexposed outcome distributions within each subgroup. Error bars indicate 95% CIs derived from 500 bootstrap replicates. "Selection Frequency" indicates the proportion of bootstrap replicates in which the variable was retained as a robust independent effect modifier in the multivariable variable selection model. Adjusted $\beta$ coefficients represent the median linear contribution of each baseline characteristic to the treatment effect size; unlike the stratified sCATT estimates, these coefficients isolate the independent contribution of each variable by accounting for correlations between baseline covariates.

*Abbreviations: BP, Blood Pressure; sCATT, scalar Conditional Average Treatment Effect on the Treated; BMI, Body Mass Index; CI, Confidence Interval.*

**A**

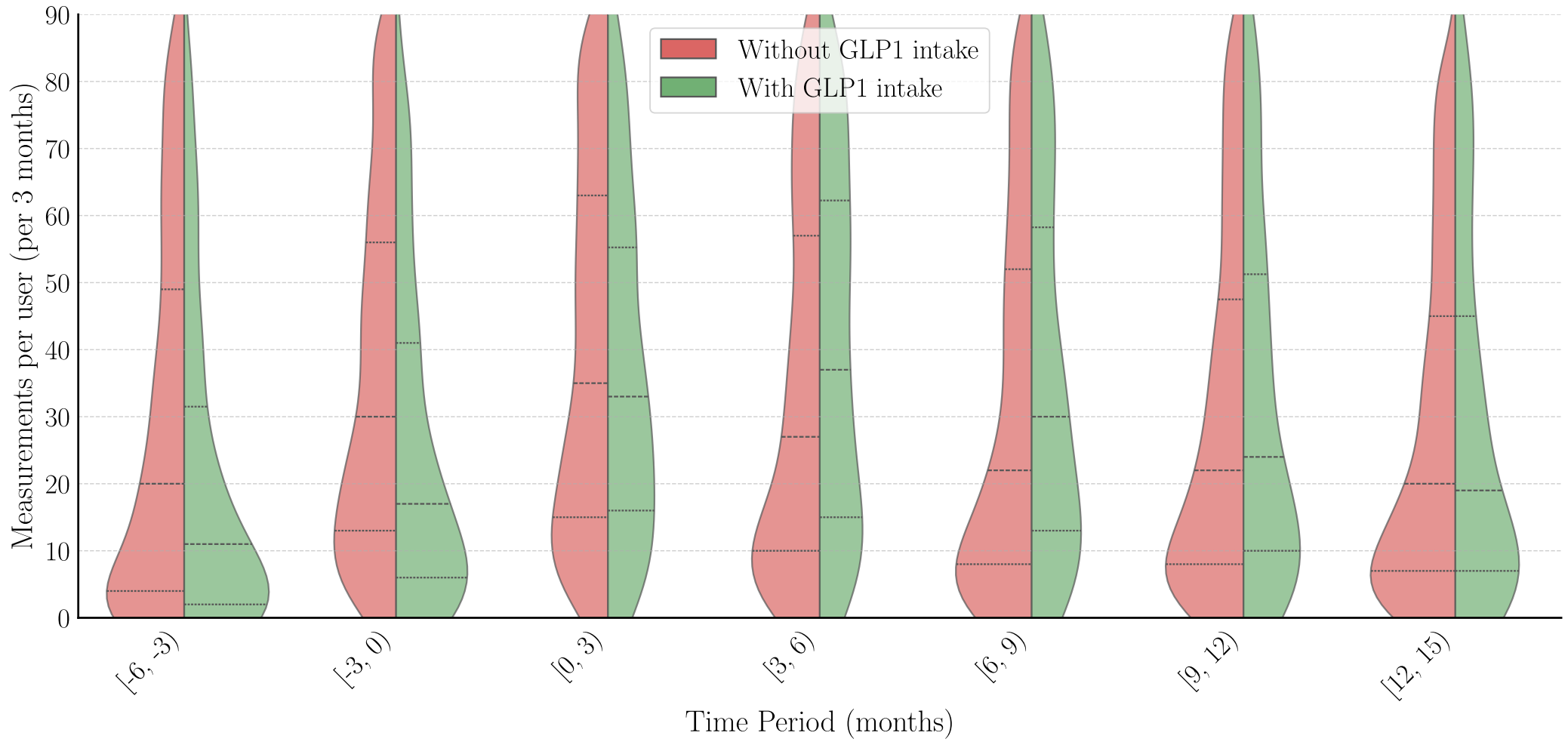


**B**

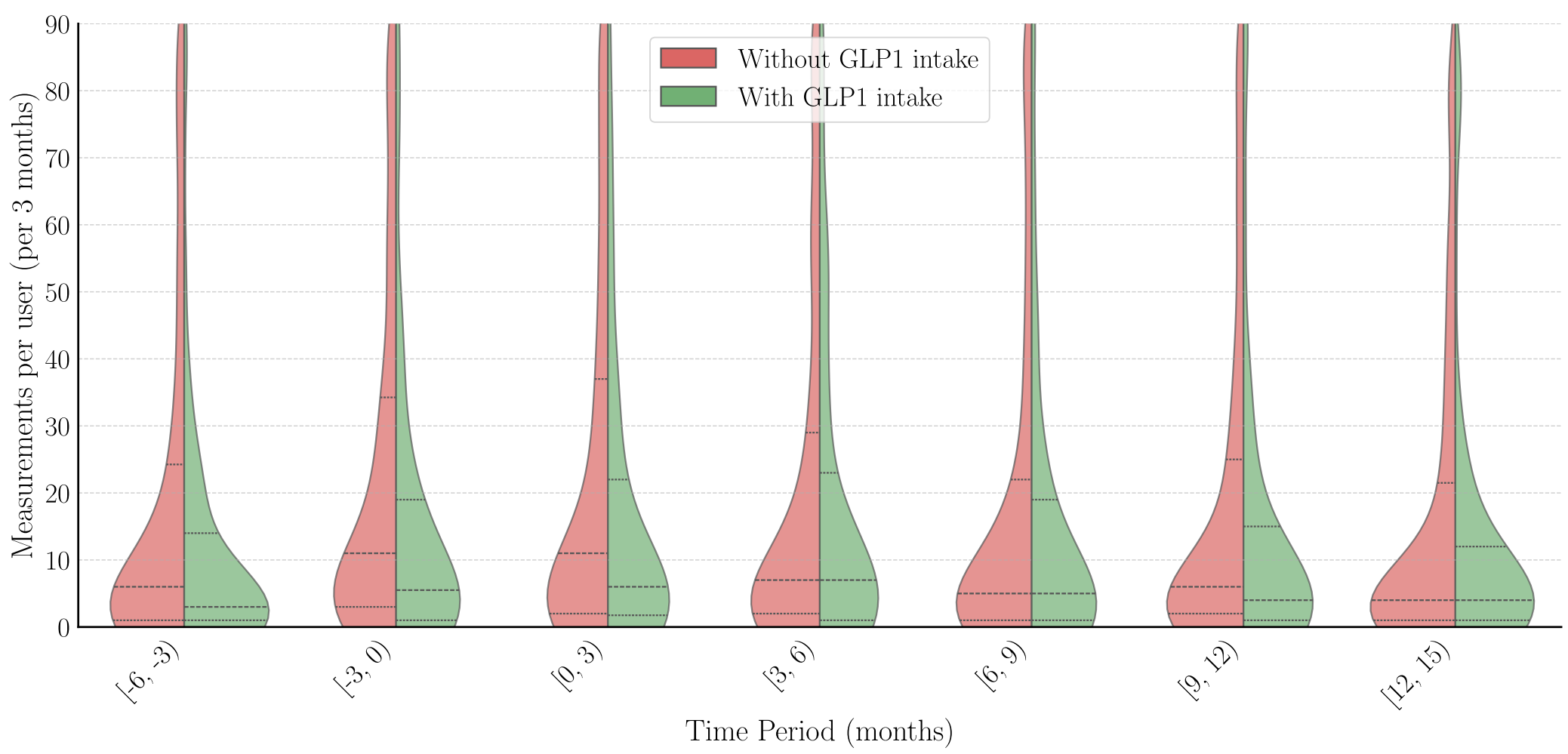


**Figure S3: Temporal density of measurements for exposed and unexposed users.** Violin plots illustrating the distribution of measurement frequency (measurements per user per 3-month interval) over the study period. **A:** Cohort 1 (Body Composition). **B:** Cohort 2 (Blood Pressure). Green distributions represent exposed users; red distributions represent unexposed users.

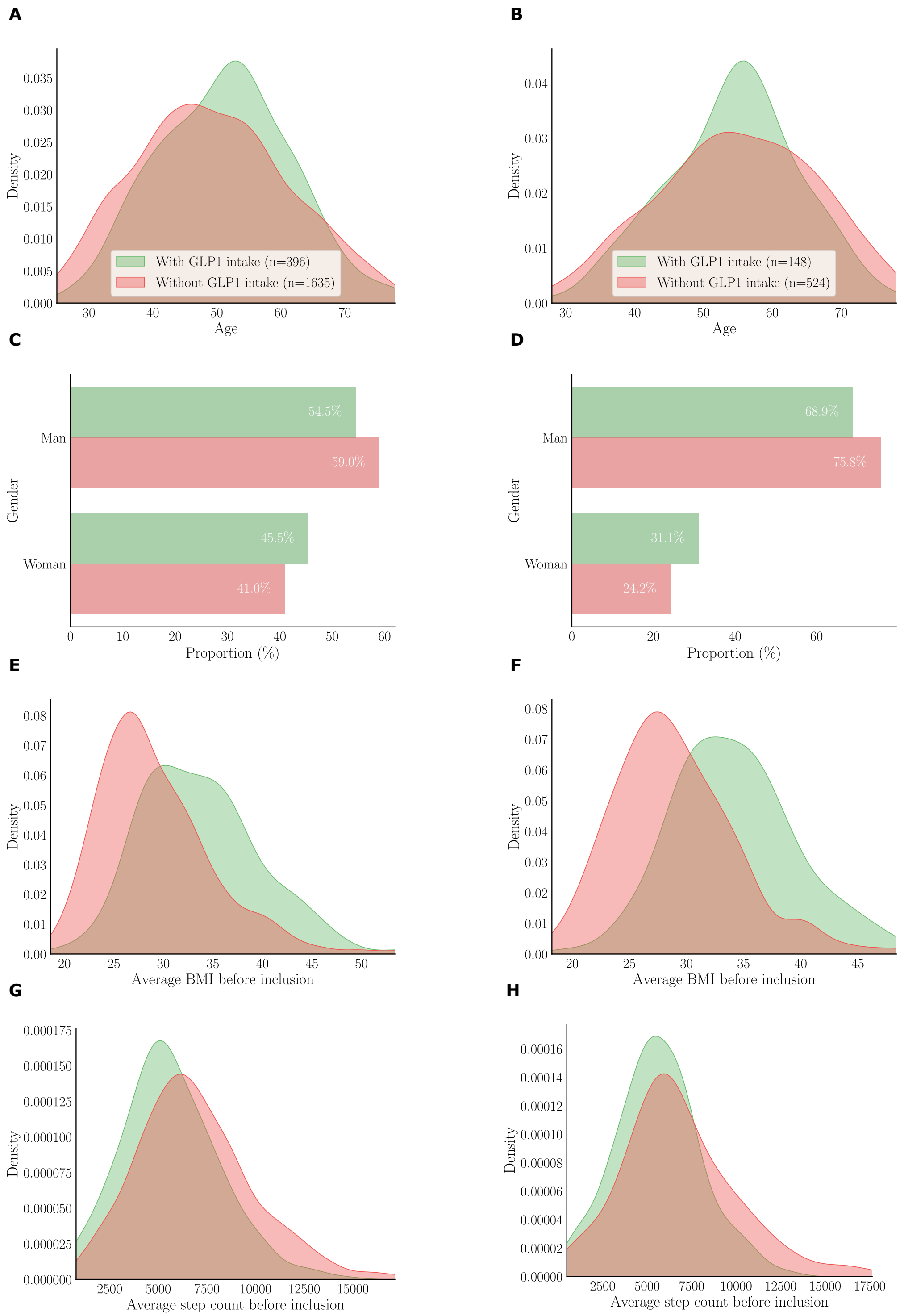


**Figure S4: Distribution of baseline characteristics prior to matching.** Density plots comparing the raw, unadjusted distributions of baseline covariates between exposed (green) and unexposed (red) users for cohort 1 (Body Composition; A, C, E, G) and cohort 2 (Blood Pressure; B, D, F, H). **A, B:** Age distribution. **C, D:** Gender proportion. **E, F:** Average BMI before inclusion. **G, H:** Average daily step count before inclusion. These plots visualize the baseline scalar confounding by indication (e.g., higher initial BMI in the exposed group) that necessitated adjustment.
*Abbreviations: BMI, Body Mass Index.*

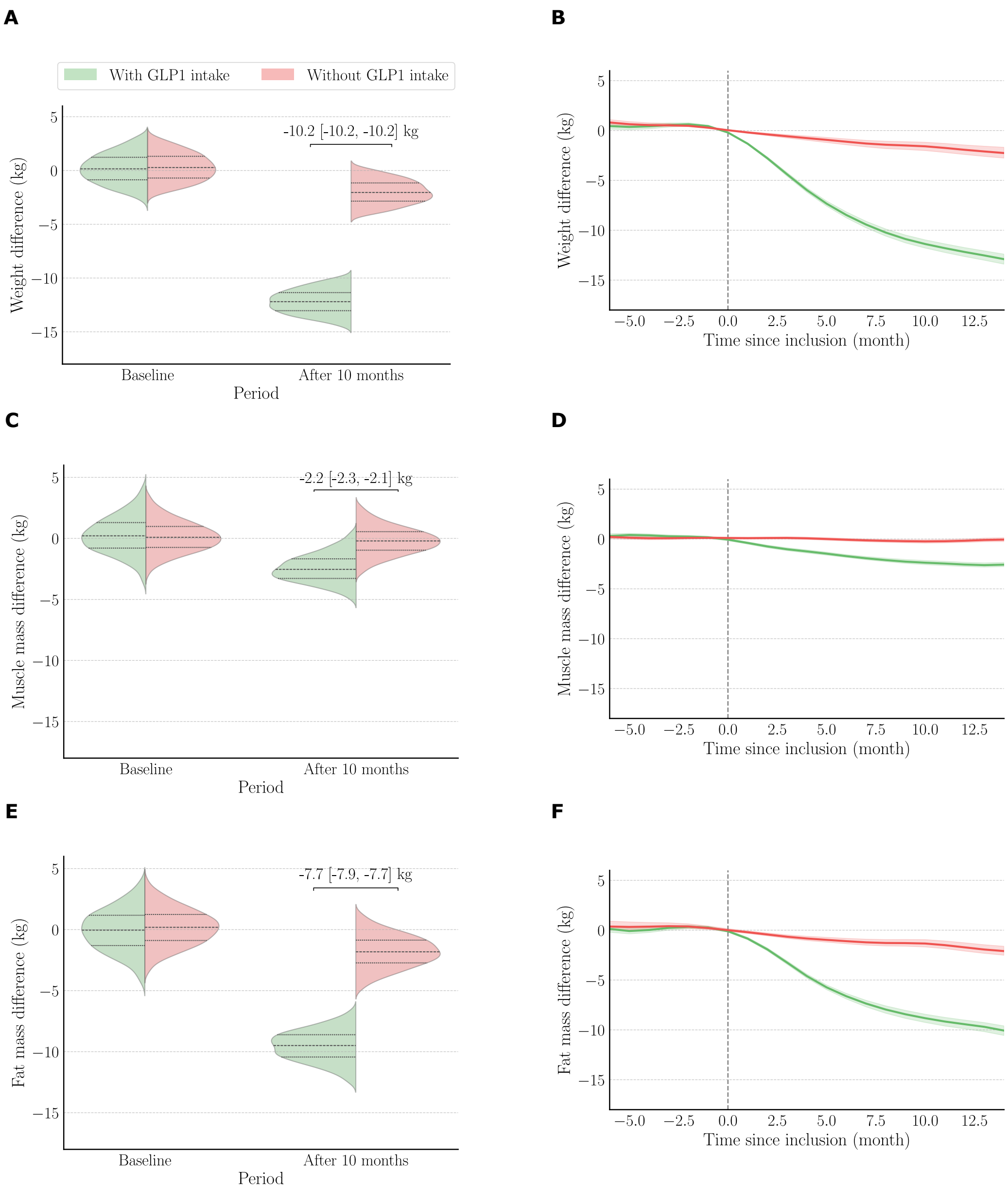


**Figure S5: Absolute distributional and longitudinal effects of GLP-1 RA initiation on body composition (kg). A, C, E:** Plots displaying the distributions barycentres of values of absolute change in total body weight (A), device-estimated muscle mass (C), and fat mass (E) in kilograms across the "Baseline" period (the pre-inclusion window of 6 months) and the "After 10 months" 4-month period (the 1-year efficacy window—months 10–14). The left side (green) represents the barycentre distribution in the exposed group, while the right side (red) represents the barycentre distribution in the matched unexposed group (counterfactuals). The bracketed values display the associated scalar Average Treatment Effect on the Treated (sATT) estimate, the estimated difference between the median of the two barycentre distributions, with 95% bootstrap CIs. **B, D, F:** Longitudinal cross-sectional mean trajectories of absolute change in total body weight (B), device-estimated muscle mass (D), and fat mass (F) in kilograms from 6 months prior to 14 months post-inclusion ($t_0$). Trajectories were reconstructed using Functional Data Analysis via Principal Component Analysis through Conditional Expectation (PACE). The green line represents the exposed group, and the red line represents the matched unexposed group (counterfactual). Shaded areas indicate 95% bootstrap pointwise CIs.
*Abbreviations: GLP-1 RA, Glucagon-Like Peptide-1 Receptor Agonist; sATT, scalar Average Treatment Effect on the Treated; CI, Confidence Interval; PACE, Principal Component Analysis through Conditional Expectation.*

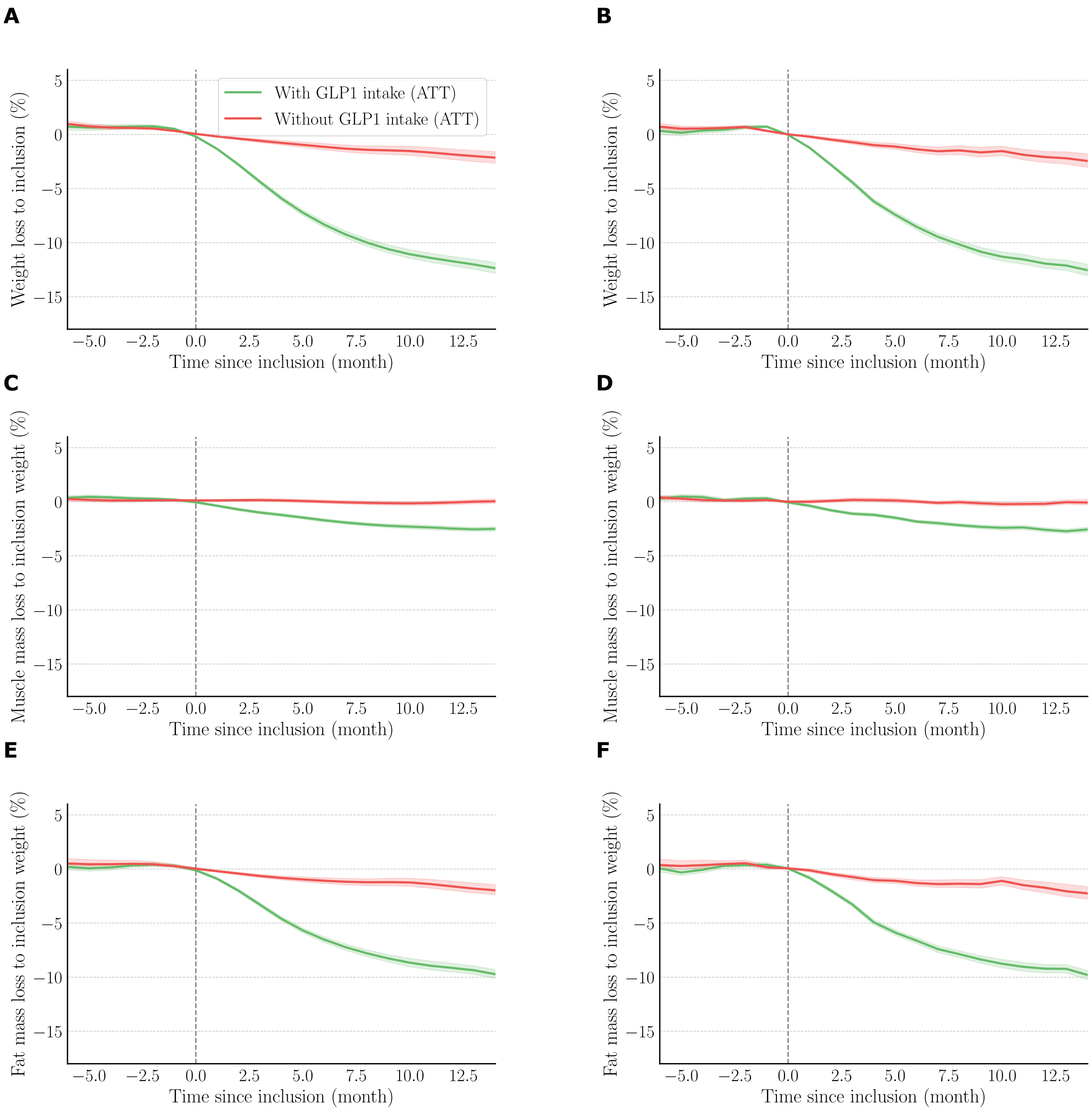


**Figure S6: Comparison of smoothed versus raw longitudinal trajectories. A, C, E:** Longitudinal cross-sectional mean trajectories of percentage change in total body weight (A), device-estimated muscle mass (C), and fat mass (E) relative to inclusion weight, estimated using the primary PACE smoothing method (identical to Figure 3). **B, D, F:** Corresponding trajectories calculated using raw, non-smoothed empirical means of the daily measurements. The green line represents the exposed group, and the red line represents the matched unexposed group.

*Abbreviations: PACE, Principal Component Analysis through Conditional Expectation.*

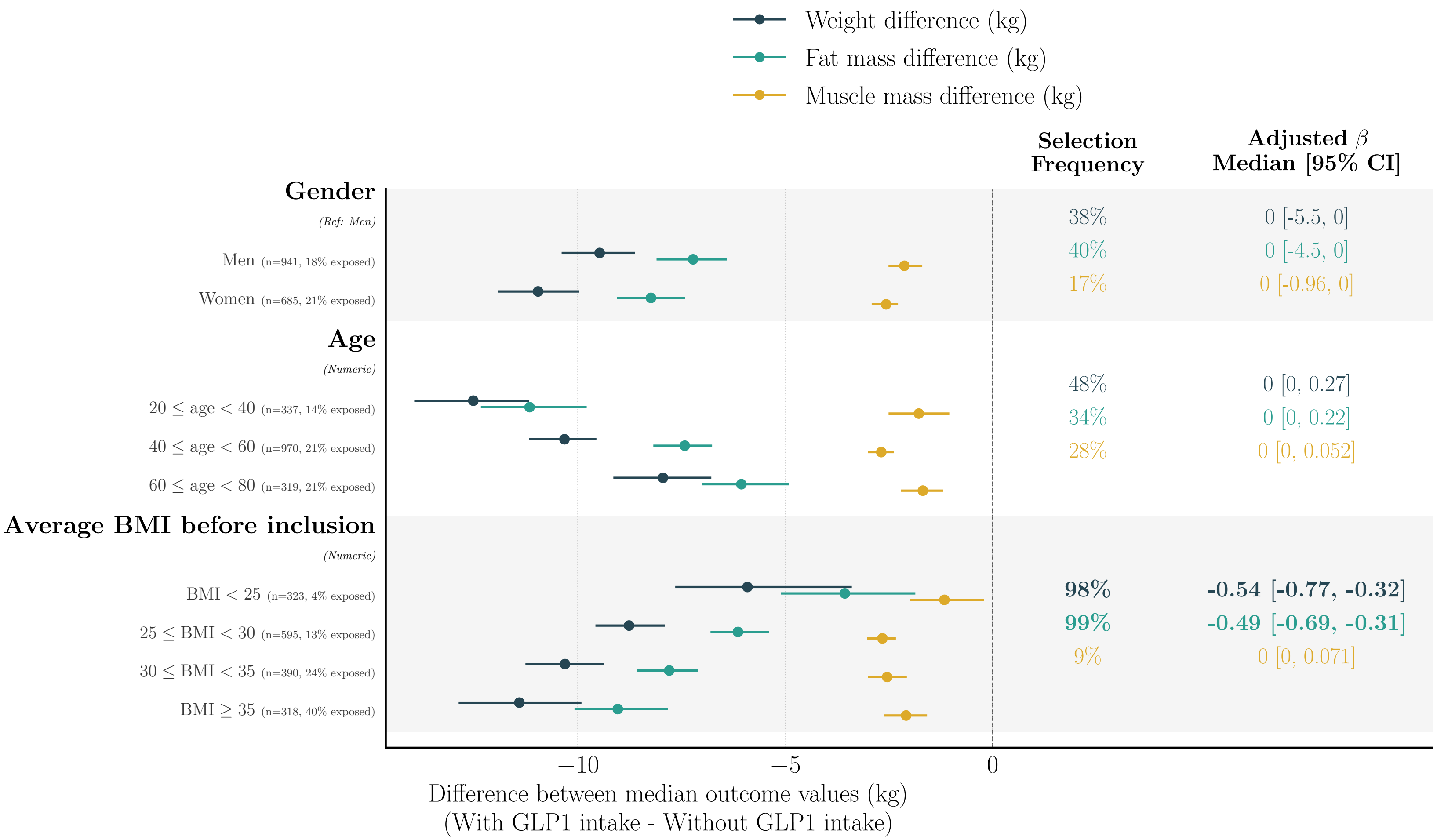


**Figure S7: Heterogeneity of treatment effect on absolute weight and body composition changes (kg).** Forest plot displaying the scalar Conditional Average Treatment Effect on the Treated (sCATT) on absolute weight loss, device-estimated fat mass loss, and muscle mass loss (kg) across stratifications of gender, age, and baseline BMI. Points represent the median difference between the barycentre distributions of the exposed and unexposed outcome distributions within each subgroup. Error bars indicate 95% CIs derived from 500 bootstrap replicates. "Selection Frequency" indicates the proportion of bootstrap replicates in which the variable was retained as a robust independent effect modifier in the multivariable selection model. Adjusted $\beta$ coefficients represent the median linear contribution of each baseline characteristic to the treatment effect size; unlike the stratified sCATT estimates, these coefficients isolate the independent contribution of each variable by accounting for correlations between baseline covariates.

*Abbreviations: sCATT, scalar Conditional Average Treatment Effect on the Treated; BMI, Body Mass Index; CI, Confidence Interval.*

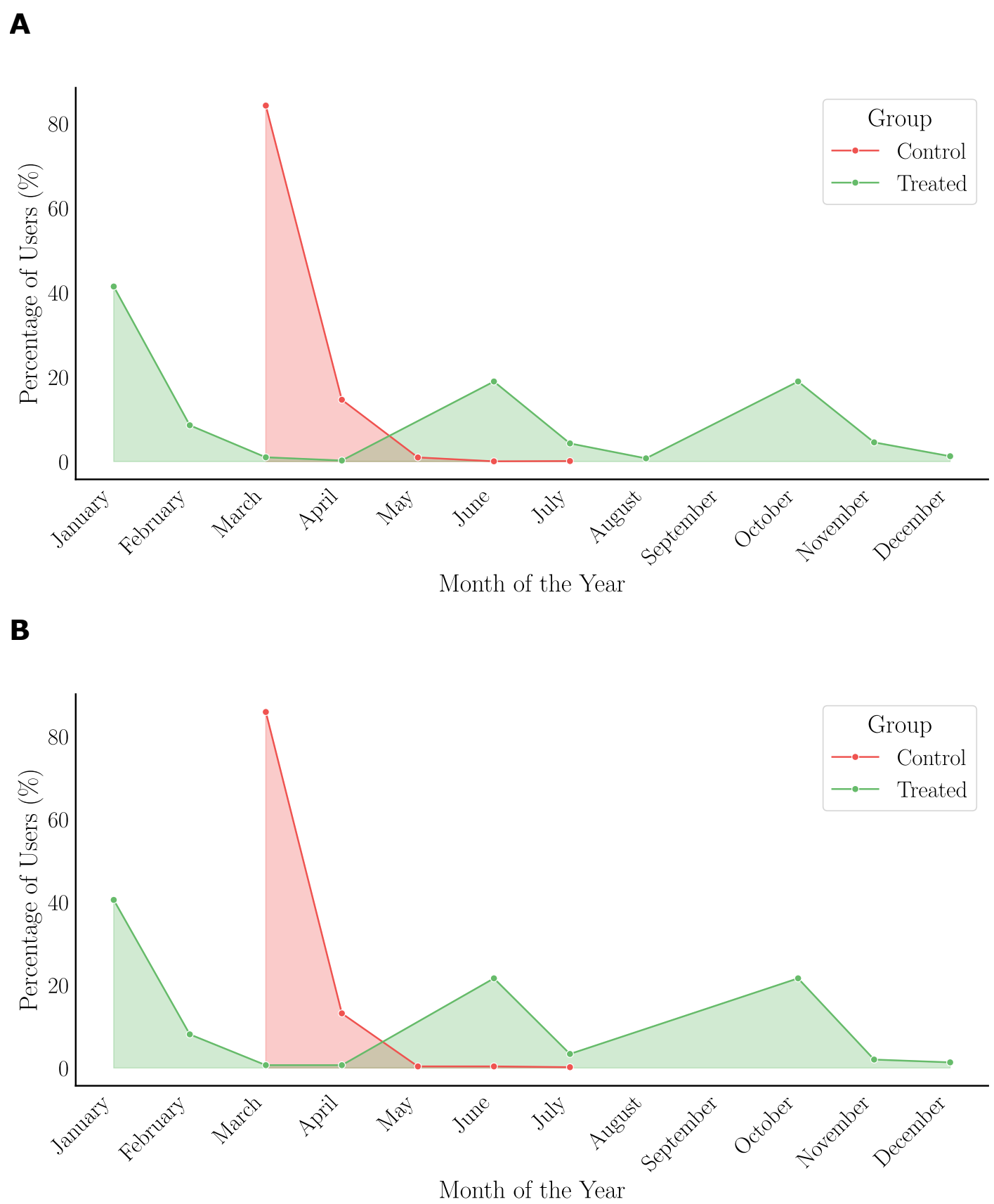


**Figure S8: Seasonal distribution of inclusion dates ($t_0$).** Histograms showing the calendar month of inclusion ($t_0$) for exposed (green) and unexposed (red) users. **A:** Cohort 1 (Body Composition). **B:** Cohort 2 (Blood Pressure). For unexposed users, $t_0$ is defined by the survey submission date, resulting in a specific seasonal clustering (primarily March). For exposed users, $t_0$ is imputed based on self-reported treatment duration categories, resulting in a more uniform distribution across the calendar year.

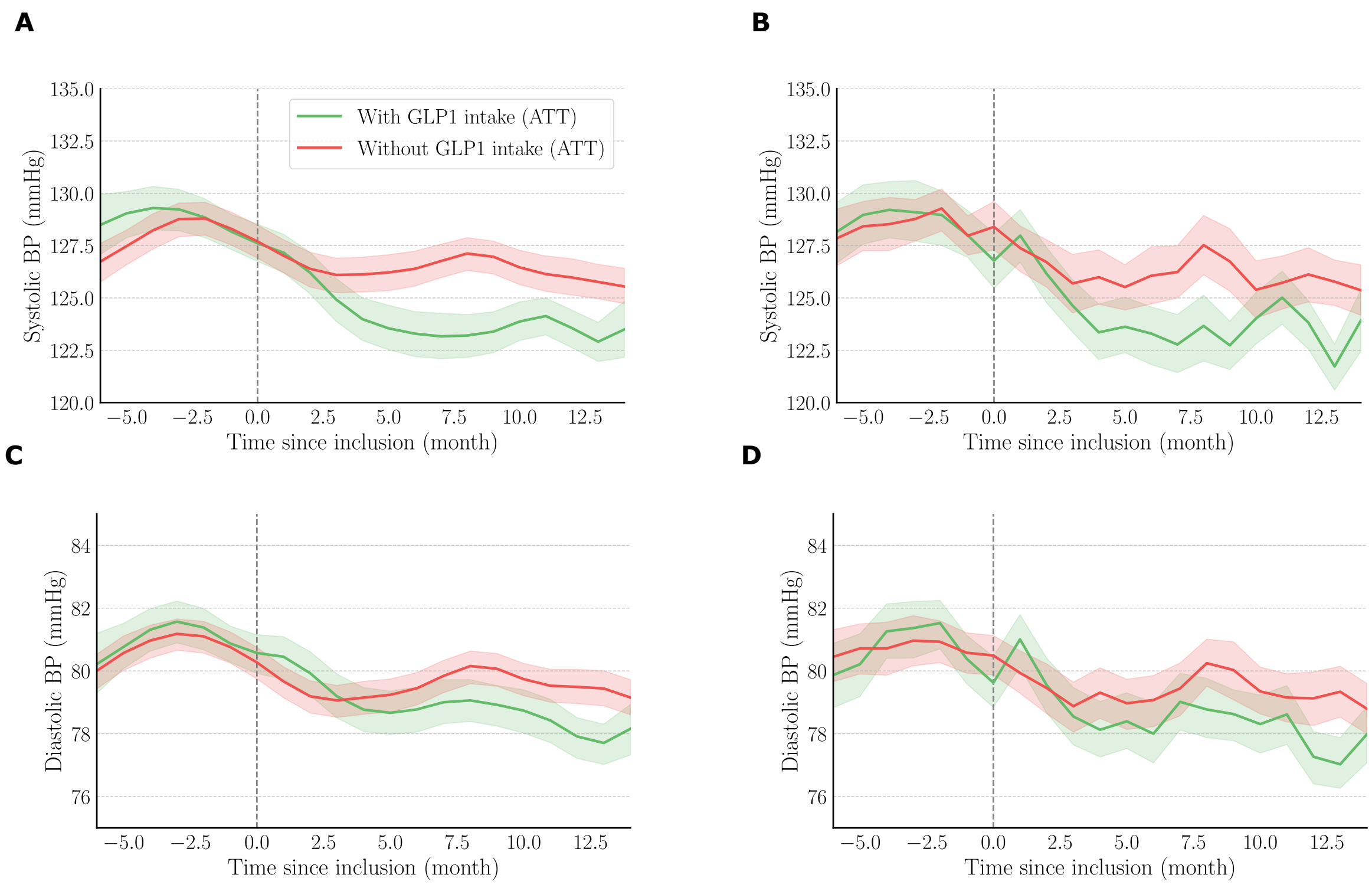


**Figure S9: Comparison of smoothed versus raw longitudinal blood pressure trajectories.** **A, C:** Longitudinal cross-sectional mean trajectories of systolic (A) and diastolic (C) blood pressure estimated using the primary PACE smoothing method (identical to Figure 5). **B, D:** Corresponding trajectories calculated using raw, non-smoothed empirical means of the daily measurements. The green line represents the exposed group, and the red line represents the matched unexposed group.
*Abbreviations: PACE, Principal Component Analysis through Conditional Expectation.*

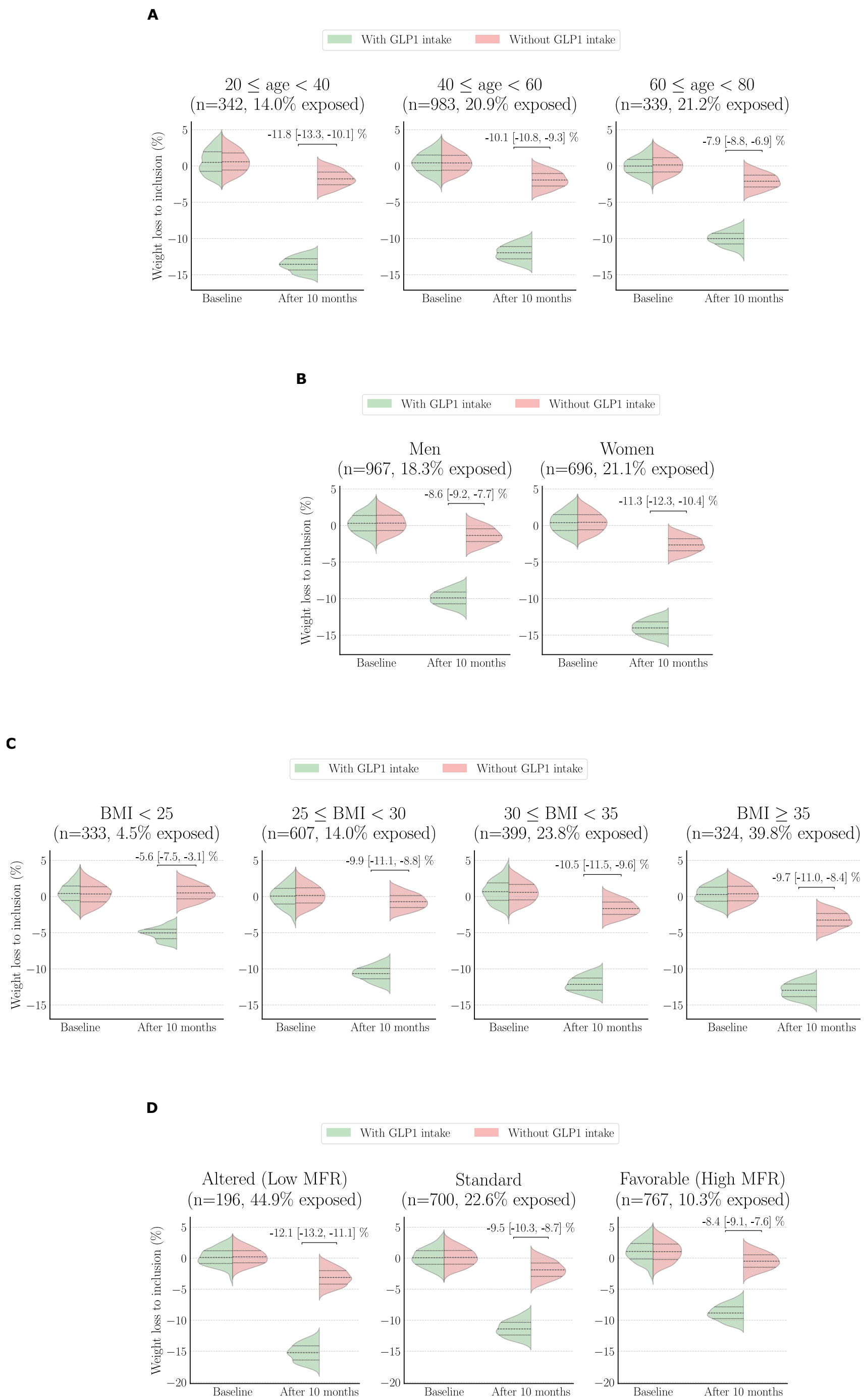


**Figure S10: Heterogeneity of treatment effect on weight loss (%) by baseline characteristics.** Barycentres of distributions stratified by age, gender, and baseline BMI. The left side (green) represents the barycentre distribution in the exposed group, and the right side (red) represents the barycentre distribution in the matched unexposed group (counterfactuals). The bracketed values display the associated scalar Conditional Average Treatment Effect on the Treated (sCATT) estimate, the estimated difference between the median of the two barycentre distributions, with 95% bootstrap CIs.

*Abbreviations: sCATT, scalar Conditional Average Treatment Effect on the Treated; BMI, Body Mass Index; CI, Confidence Interval.*

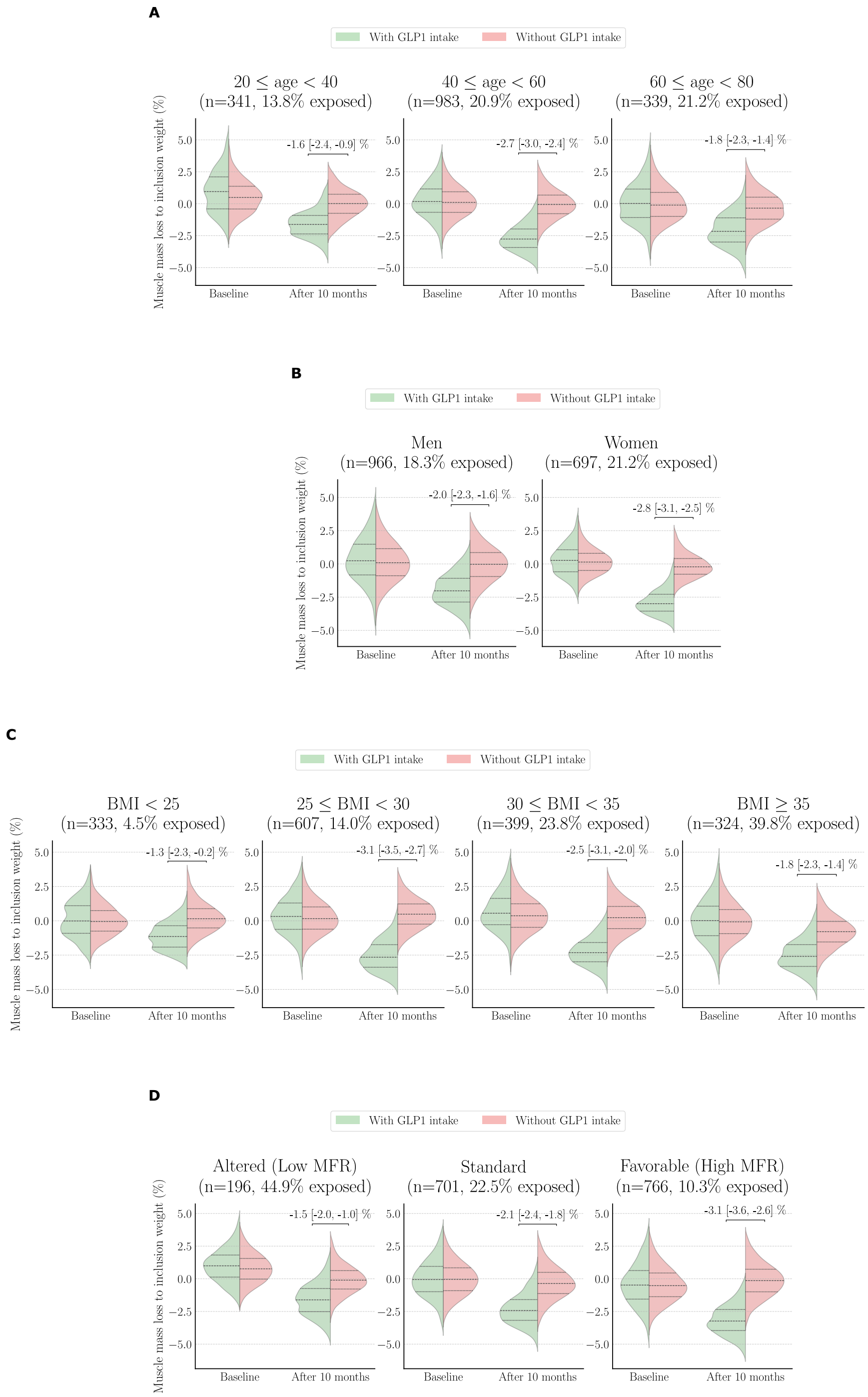


**Figure S11: Heterogeneity of treatment effect on device-estimated muscle mass loss (%) by baseline characteristics.** Barycentres of distributions stratified by age, gender, baseline BMI, and baseline muscle-to-fat ratio. The left side (green) represents the barycentre distribution in the exposed group, and the right side (red) represents the barycentre distribution in the matched unexposed group (counterfactuals). The bracketed values display the associated scalar Conditional Average Treatment Effect on the Treated (sCATT) estimate, the estimated difference between the median of the two barycentre distributions, with 95% bootstrap CIs.
*Abbreviations: sCATT, scalar Conditional Average Treatment Effect on the Treated; BMI, Body Mass Index; CI, Confidence Interval.*

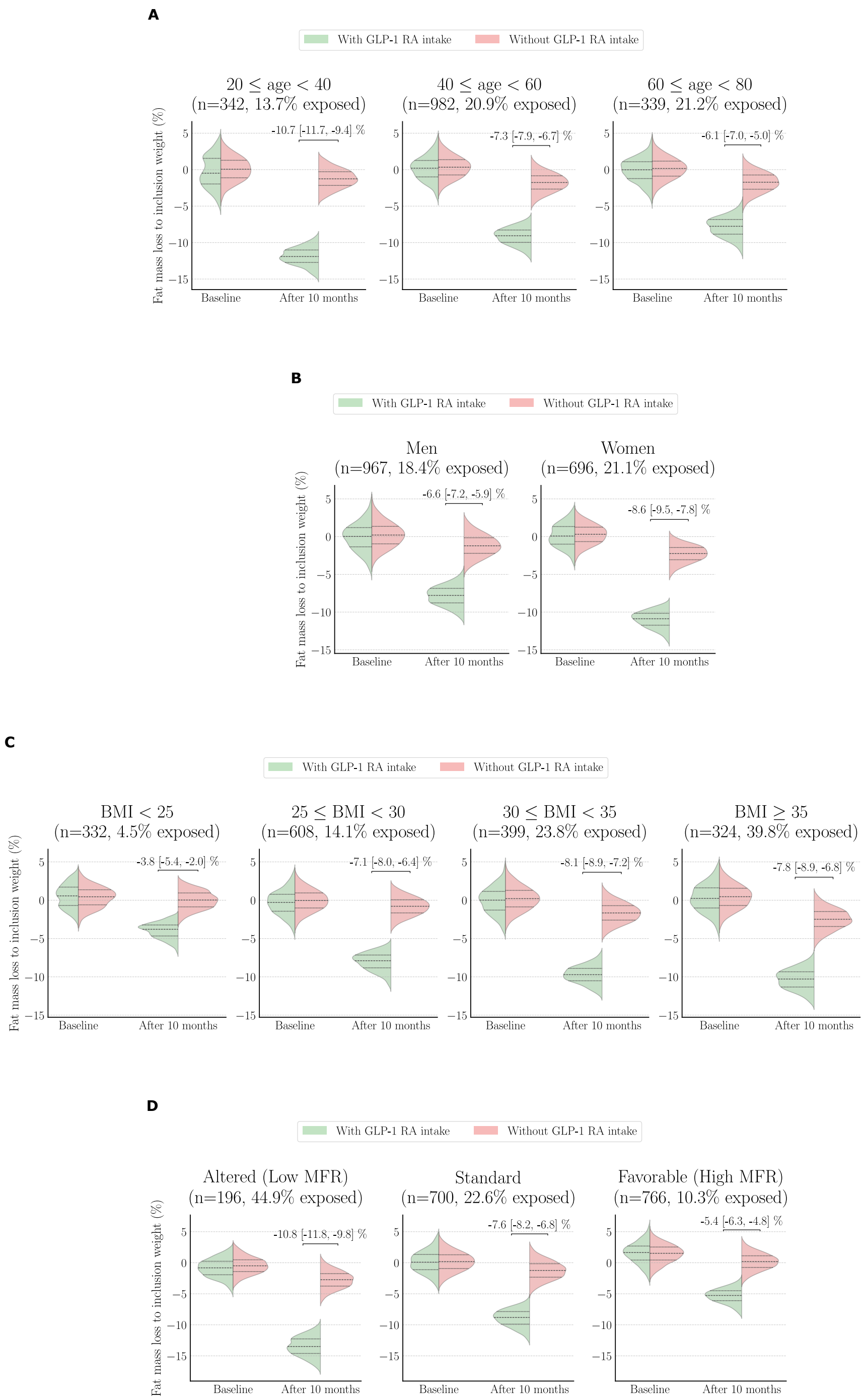


**Figure S12: Heterogeneity of treatment effect on device-estimated fat mass loss (%) by baseline characteristics.** Barycentres of distributions stratified by age, gender, baseline BMI, and baseline muscle-to-fat ratio. The left side (green) represents the barycentre distribution in the exposed group, and the right side (red) represents the barycentre distribution in the matched unexposed group (counterfactuals). The bracketed values display the associated scalar Conditional Average Treatment Effect on the Treated (sCATT) estimate, the estimated difference between the median of the two barycentre distributions, with 95% bootstrap CIs.
*Abbreviations: sCATT, scalar Conditional Average Treatment Effect on the Treated; BMI, Body Mass Index; CI, Confidence Interval.*

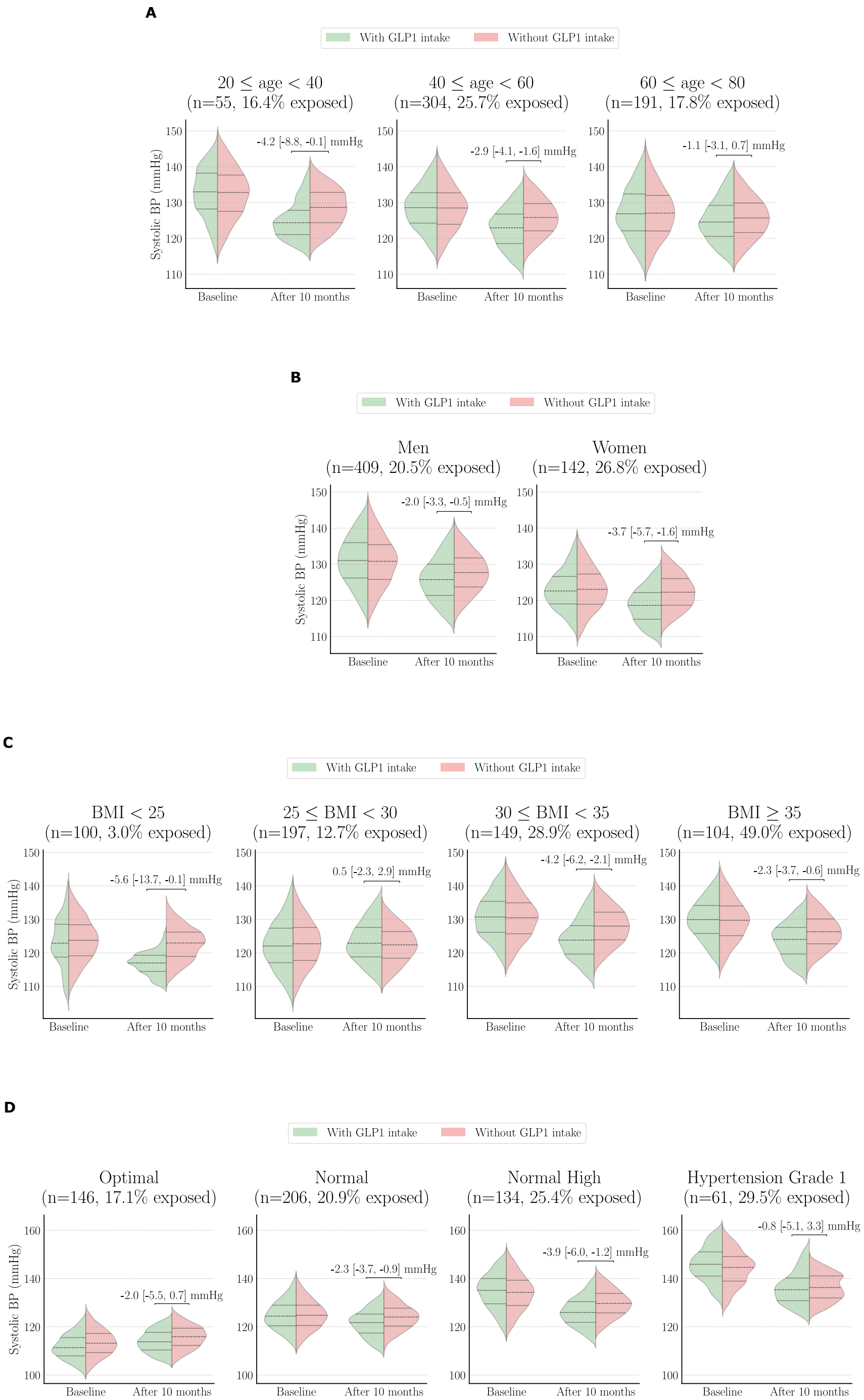


**Figure S13: Heterogeneity of treatment effect on systolic blood pressure by baseline characteristics.** Barycentres of distributions stratified by age, gender, baseline BMI, and baseline systolic BP category. The left side (green) represents the barycentre distribution in the exposed group, and the right side (red) represents the barycentre distribution in the matched unexposed group (counterfactuals). The bracketed values display the associated scalar Conditional Average Treatment Effect on the Treated (sCATT) estimate, the estimated difference between the median of the two barycentre distributions, with 95% bootstrap CIs.
*Abbreviations: sCATT, scalar Conditional Average Treatment Effect on the Treated; BMI, Body Mass Index; BP, Blood Pressure; CI, Confidence Interval.*

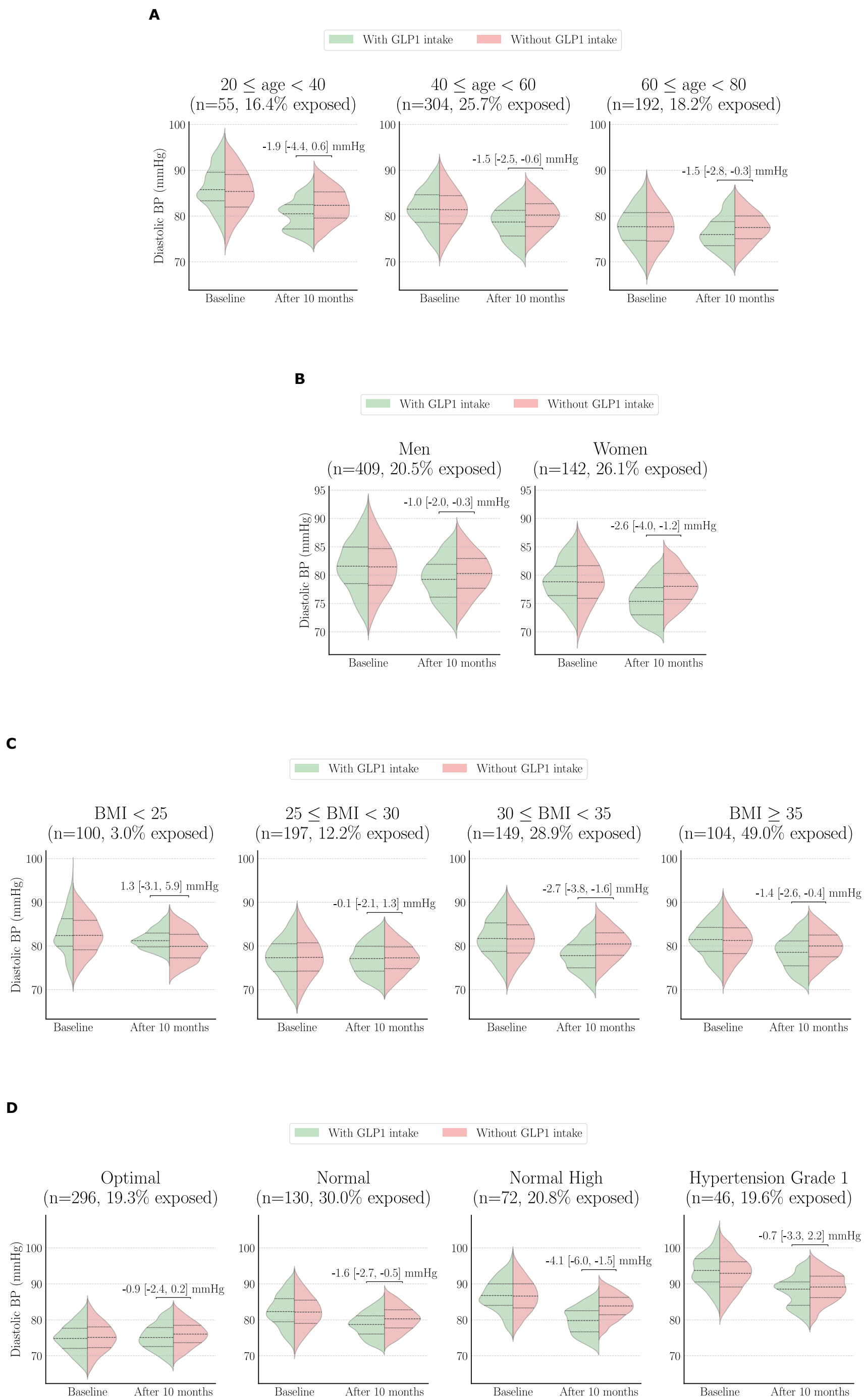


**Figure S14: Heterogeneity of treatment effect on diastolic blood pressure by baseline characteristics.** Barycentres of distributions stratified by age, gender, baseline BMI, and baseline systolic BP category. The left side (green) represents the barycentre distribution in the exposed group, and the right side (red) represents the barycentre distribution in the matched unexposed group (counterfactuals). The bracketed values display the associated scalar Conditional Average Treatment Effect on the Treated (sCATT) estimate, the estimated difference between the median of the two barycentre distributions, with 95% bootstrap CIs.

*Abbreviations: sCATT, scalar Conditional Average Treatment Effect on the Treated; BMI, Body Mass Index; BP, Blood Pressure; CI, Confidence Interval.*

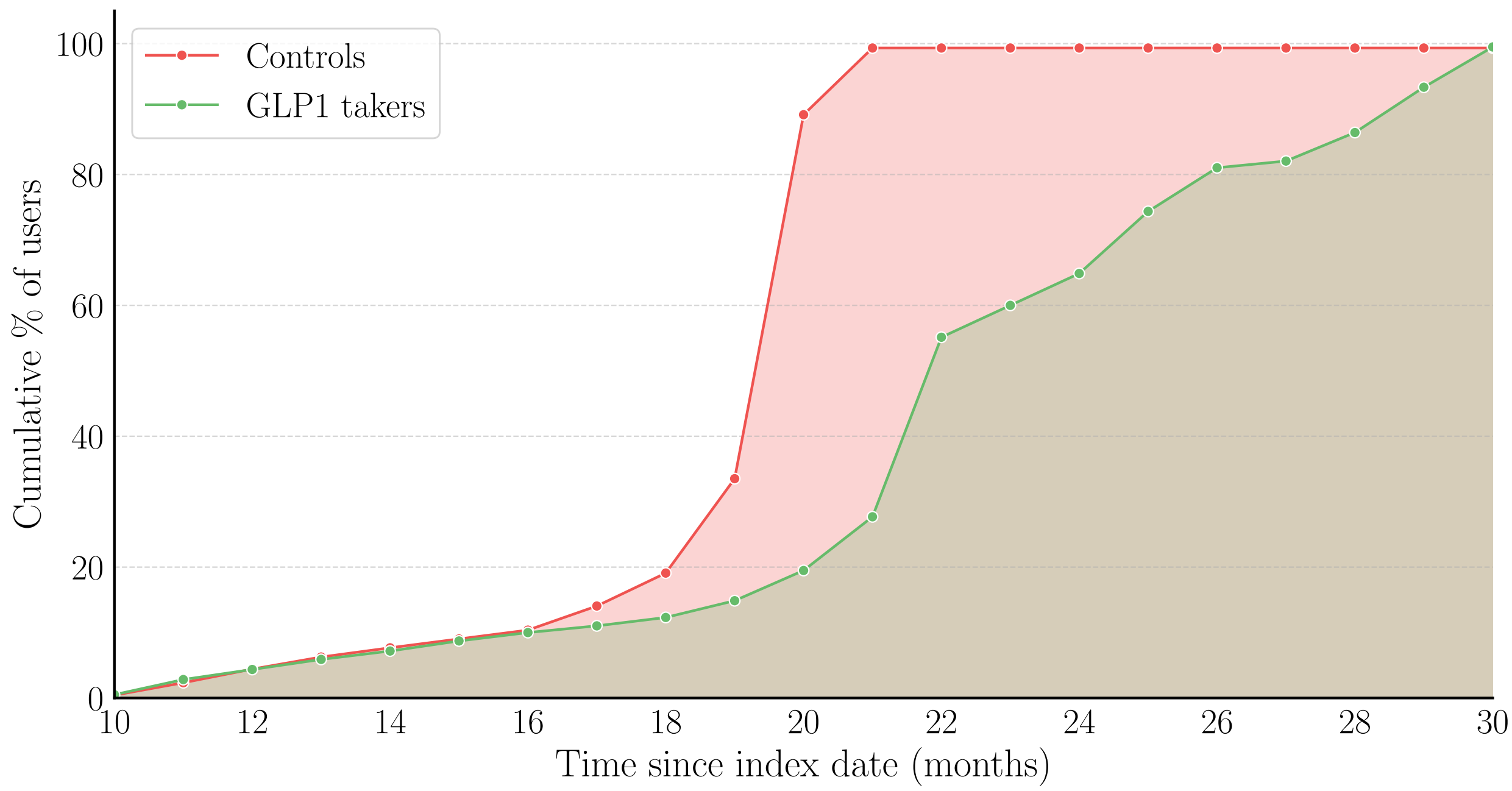


**Figure S15: Cumulative distribution of last measurement time since index date ($t_0$) for Cohort 1** This figure illustrates the longitudinal durability of user engagement in Cohort 1. The x-axis represents the duration in months from the index date to the user's final recorded weight measurement. The divergence between the groups, particularly after month 20, indicates significantly higher retention among GLP-1 RA initiators, with approximately 40% of users maintaining active monitoring beyond 24 months and through the 30-month study horizon. This sustained, self-motivated engagement serves as a proxy for the long-term feasibility and acceptability of digital health monitoring in this patient population.
*Abbreviations: GLP-1 RA, Glucagon-Like Peptide-1 Receptor Agonist.*
